# A graph-based evidence synthesis approach to detecting outbreak clusters: An application to dog rabies

**DOI:** 10.1371/journal.pcbi.1006554

**Published:** 2018-12-17

**Authors:** Anne Cori, Pierre Nouvellet, Tini Garske, Hervé Bourhy, Emmanuel Nakouné, Thibaut Jombart

**Affiliations:** 1 MRC Centre for Outbreak Analysis and Modelling, Department of Infectious Disease Epidemiology, School of Public Health, Imperial College London, London, United Kingdom; 2 School of Life Sciences, University of Sussex, Brighton, United Kingdom; 3 Unit Lyssavirus Dynamics and Host Adaptation, WHO Collaborating Centre for Reference and Research on Rabies, Institut Pasteur, Paris, France; 4 Département fièvres hémorragiques virales, Institut Pasteur de Bangui, Bangui, République Centrafricaine; University of Chicago, UNITED STATES

## Abstract

Early assessment of infectious disease outbreaks is key to implementing timely and effective control measures. In particular, rapidly recognising whether infected individuals stem from a single outbreak sustained by local transmission, or from repeated introductions, is crucial to adopt effective interventions. In this study, we introduce a new framework for combining several data streams, e.g. temporal, spatial and genetic data, to identify clusters of related cases of an infectious disease. Our method explicitly accounts for underreporting, and allows incorporating preexisting information about the disease, such as its serial interval, spatial kernel, and mutation rate. We define, for each data stream, a graph connecting all cases, with edges weighted by the corresponding pairwise distance between cases. Each graph is then pruned by removing distances greater than a given cutoff, defined based on preexisting information on the disease and assumptions on the reporting rate. The pruned graphs corresponding to different data streams are then merged by intersection to combine all data types; connected components define clusters of cases related for all types of data. Estimates of the reproduction number (the average number of secondary cases infected by an infectious individual in a large population), and the rate of importation of the disease into the population, are also derived. We test our approach on simulated data and illustrate it using data on dog rabies in Central African Republic. We show that the outbreak clusters identified using our method are consistent with structures previously identified by more complex, computationally intensive approaches.

## Author summary

Early assessment of infectious disease outbreaks is key to implementing timely and effective control measures. In particular, rapidly recognising whether infected individuals stem from a single outbreak sustained by local transmission, or from repeated introductions, is crucial to adopt effective interventions. In this study, we introduce a new approach which combines different types of data to identify clusters of related cases of an infectious disease. This approach relies on representing each type of data (e.g. temporal, spatial, or genetic) as a graph where nodes are cases, and two nodes are connected if the corresponding cases are closely related for this data. Our method then identifies clusters of cases which likely stem from the same introduction. Furthermore, we can use the size of these clusters to infer transmissibility of the disease and the number of importations of the pathogen into the population. We apply this approach to analyse dog rabies epidemics in Central African Republic. We show that outbreak clusters identified using our method are consistent with structures previously identified by more complex and computationally intensive approaches. Using simulated rabies epidemics, we show that our method has excellent potential for optimally detecting outbreak clusters. We also identify promising areas of research for transforming our method into a routine analysis tool for processing disease surveillance data.

## Introduction

Infectious disease outbreaks are a recurring threat to humans and animals, with potentially disastrous impacts on human health, economy, and biodiversity. Over the last decade, major epidemics such as the 2009 influenza pandemic [1], the emergence of the Middle-East Respiratory Syndrome (MERS, [2,3]) and the West-African Ebola virus disease outbreak (EVD, [4,5]) have re-emphasized the need for assessing outbreaks at an early stage. Indeed, rapid identification of clusters of cases linked by transmission and subsequent intervention remain our best chance of containing, or at least mitigating disease epidemics.

The identification of clusters of cases is also key to adapting the response in ongoing epidemics. Indeed, early assessment of the relative contributions of local transmission versus case importation is essential for designing appropriate intervention strategies. For instance, a nosocomial outbreak may be driven by within-hospital transmissions or repeated introductions from the community, calling for different control measures [6,7]. Similarly at a country level, local transmissions and importations of cases from other countries will require different control measures, *e*.*g*. social distancing and prevention *versus* border closing [8,9]. In the case of zoonotic infections, it is also crucial to identify the extent to which within-species transmission and spill-over from the reservoir (i.e. cross-species transmission) contribute to the observed incidence, as illustrated in the case of avian influenza [10,11], bovine tuberculosis [12], or MERS [3,8,9].

Methodologically, the identification of clusters of cases linked by transmission (i.e. cases belonging to the same transmission tree or stemming from a single introduction, here referred to as 'outbreak clusters’) is strongly related to other fields which have received considerable attention from statisticians and modellers over the last decades. First, it is closely linked to outbreak detection methods, which generally aim to identify excesses of cases compared to a reference ‘baseline’ in incidence time series [13–19], and are for instance routinely used to detect the beginning of seasonal influenza epidemics from surveillance data [20–22]. Some extensions to spatiotemporal data [23–26] have shown geographic information can be a useful complement to temporal data [27,28], but little efforts have been devoted to generalising these approaches to other types of data such as pathogen whole genome sequences (WGS). Second, the identification of outbreak clusters is also closely related to outbreak reconstruction methods, which infer transmission chains using complex outbreak models integrating multiple sources of data such as time, space, and WGS [29–32]. Lastly, population genetics has had a long-standing tradition of developing clustering methods [33–35], some of which have proved useful for studying pathogen populations [36,37]. Unfortunately, despite these connections to well-developed methodological fields, the integration of multiple data sources (e.g. epidemiological and genomic data) to identify outbreak clusters remains in its infancy [38].

**Fig 1:**
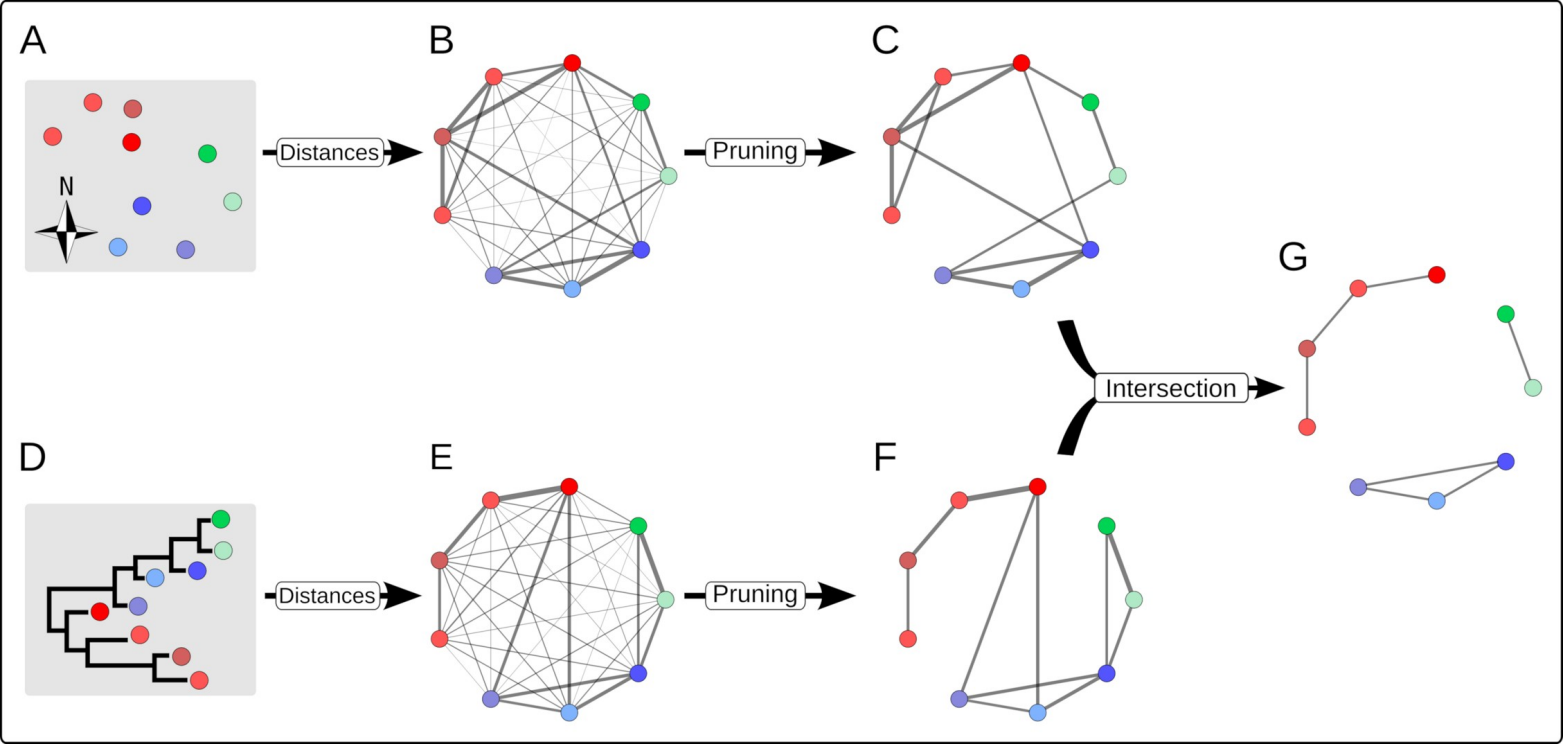
Schematic illustration of our graph approach for combining multiple data streams to identify outbreak clusters. In this example, two data streams are considered: the spatial locations of the cases (A) and a phylogeny of pathogens from these cases (D). The three ‘actual’ outbreak clusters are identified in red, blue and green, using different shadings to identify individual cases. Each data source defines a fully connected graph where nodes represent cases and edges are weighted by the spatial (B) and genetic distances (E). Thicker edges represent smaller weights (distances) between cases. Each graph is then pruned separately, removing edges whose weight exceeds a given cutoff (C, F). The intersection of these graphs defines a new graph which retains only edges present in every pruned graph (G). The resulting clusters of cases indicate likely outbreak clusters.

In this study, we introduce a new, simple and intuitive framework for combining various sources of information to identify clusters of related cases of a disease. This evidence synthesis approach can combine various data streams such as the timing and location of the cases, as well as WGS of the pathogen, to identify such outbreak clusters. Our method relies on the observation that, in an outbreak, individuals who infect one another are likely to be closely related with respect to various characteristics; for instance, their symptom onsets appear within the same time period and in neighbouring locations, and they bear genetically similar pathogen strains. We consider that, to be part of the same outbreak cluster, two cases must be closely related in all relevant data streams. These data sources describe relationships between cases in intrinsically different spaces (e.g., temporal, spatial, genetic), but they can all be used to compute pairwise distances between cases (e.g. number of days between dates of onset, geographic distance between locations, number of mutations between pathogen WGS). We define, for each data stream, a weighted graph, where nodes correspond to cases, and the edge between two cases is weighted by the pairwise distance (for this data stream) between these two cases, so that ‘heavy’ edges indicate pairs of cases unlikely to have infected one another. To retain only relevant connections, each graph is then pruned by removing heavy edges, defined as edges whose weight exceeds a predefined cutoff distance (Fig 1).

Defining the adequate cutoff is central to identifying clusters of related cases. We develop a framework for defining cutoffs based on the expected distance distributions between observed cases in an outbreak. This enables us to incorporate pre-existing information about the disease, e.g. the distribution of its serial interval (the time between symptoms onset in a case and its infector), its spatial kernel (the distribution of geographical distances between a case and its infector), and its mutation rate. Typically, such information is available for fully observed outbreaks, which is unlikely to be the case in practice. To address this issue, our approach also accounts for underreporting. We give a general solution to the expected distances between observed cases for a given level of reporting (i.e. fraction of observed cases), as well as some analytical results for common distributions used to describe distances between cases in time (the serial interval), space (the spatial kernel), and for genetic distances (the molecular clock).

Each pruned graph represents potential epidemiological links between cases for a given data source. To combine these pieces of information, we merge the pruned graphs by intersection, i.e. retaining edges present in all pruned graphs, so that the resulting connected components of the final graph defines clusters of cases related for all types of data (Fig 1). The resulting clusters identify sets of cases likely belonging to the same transmission tree. Importantly, the sizes of the clusters also contain information about the underlying transmissibility [8]. We exploit this information by deriving estimates of the reproduction number, *R* (the average number of secondary cases infected by an infectious individual in a large population), given the empirical distribution of the cluster sizes and the assumed reporting rate.

We illustrate our approach using surveillance data used for monitoring dog rabies in Bangui, the capital of Central African Republic [39]. Rabies is an important and complex neglected zoonosis mainly transmitted to humans by dogs [40,41]. Campaigns to eliminate canine rabies through the vaccination of dogs, the treatment of all potential human rabies exposures, and the improvement of education about rabies prevention are ongoing but are in many low and middle income countries of limited impact due to the lack of understanding of the dynamics of the disease [42]. In Bangui, the dynamics of rabies is likely relying on a combination of local transmission, i.e. between dogs within the city, and importations, i.e. rabid dogs entering the city from neighbouring locations, therefore providing an interesting model for a proof of concept of our approach [39]. Characterising such dynamics, and in particular the relative importance of local transmission versus importations would be critical to implementing effective control strategies. If local transmission dominates the dynamics, then mass vaccination could be sufficient for control. However, if the repeated importations dominate the dynamics, restricting these and focusing on finding and vaccinating the external ‘source(s)’ of infections would be more efficient.

Relying on dates of onset, geographic locations, and viral genetic sequences, we characterize the epidemiological situation using our method and compare the results to previous, more complex and computationally intensive approaches [39]. We also test the performance of our method by applying it to a range of simulated datasets. Results suggest our approach provides a fast, flexible, and efficient tool for detecting clusters of cases of an infectious disease linked by transmission.

## Results

**Fig 2:**
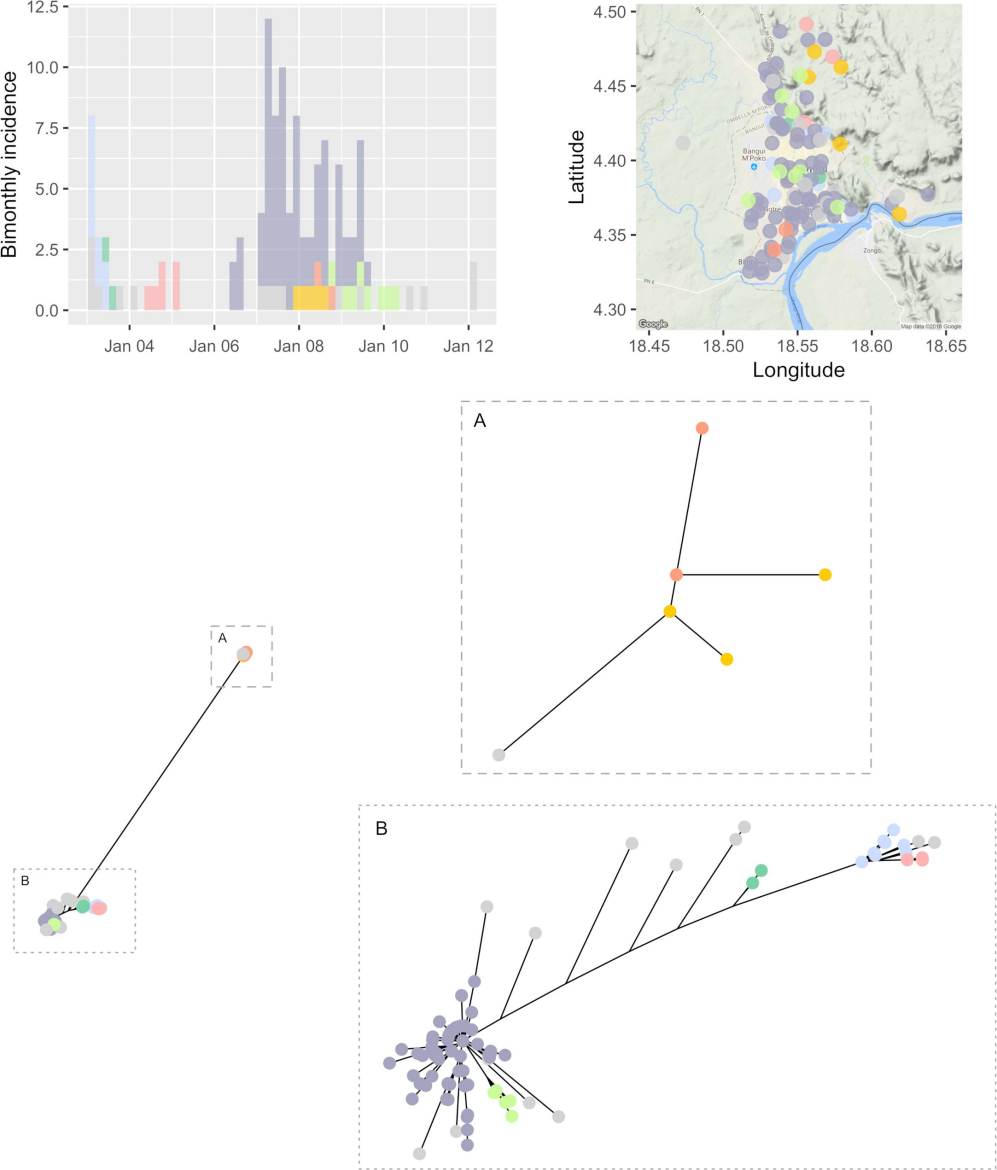
Visualization of the clusters of cases of rabies. The top left panel shows the incidence of reported cases of rabies over time, by date of report; cases identified as belonging to the same outbreak cluster (using all distances) are shown in the same colour (grey indicates singletons). The top right panel shows the geographic locations of the reported cases using the same colour coding as the incidence panel. The bottom panel shows the unrooted phylogeny obtained by Neighbour-Joining on Hamming distances (i.e. number of different nucleotides) between sampled sequences; the full tree showing two distinct strains more than 700 nucleotides apart is plotted on the left. Details of the two clades are provided in inset A) and B). A reporting rate of 20% was assumed, and pruning cutoff distances corresponding to the 95% quantiles of the input distance distributions were used (see S1 Text for sensitivity analyses on these assumptions).

**Fig 3:**
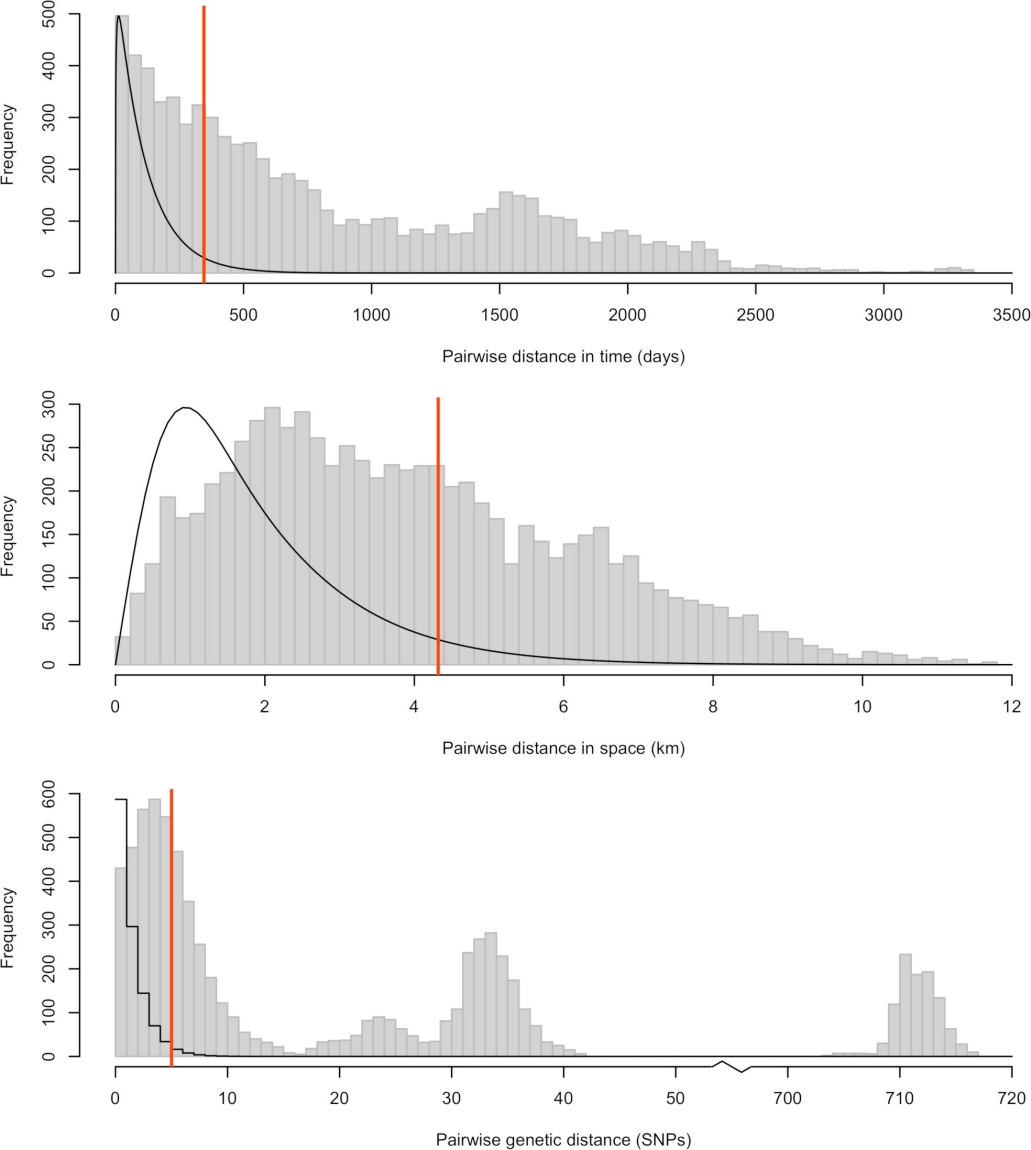
Distribution of pairwise temporal (top), spatial (middle) and genetic (bottom) distances for rabies in Bangui. The temporal distance is defined as the time between reporting of the cases. The spatial distance is defined as the Euclidean distance between the geographic locations of cases. The genetic distance is defined as the Hamming distance between the sequenced isolates. The grey histograms show the observed pairwise distances between any two cases reported in Bangui. The solid black lines show the input distribution of distances between a case and its closest observed ancestor, given an assumed reporting rate of 20% (see S1 Text for sensitivity analyses to this assumption). Distributions have been rescaled to fit on the same graph as the histograms. The red vertical lines show the cutoffs corresponding to the 95% quantiles of these distributions. For a given data stream and a given choice of cutoff, pairs of cases with observed distance above the cutoff are considered not connected, and the corresponding graph edges are removed at the pruning step (see Fig 1).

151 dogs infected with rabies were reported in Bangui, the capital of the Central African Republic between the 6th January 2003 and the 6th March 2012. Of these, 123 cases had a date of report available, as well as a geographical location and viral genome sequences (Fig 2). Pairwise distances between these 123 cases are shown in Fig 3, separately for temporal, spatial, and genetic distances. The distribution of spatial distances was unimodal, with an average pairwise distance of 3.9km. In contrast, the distribution of temporal distances exhibited two peaks, one at very short time differences, and one around 4 years, likely driven by the multiple peaks in the observed incidence time series (see Fig 2). The distribution of genetic distances was multimodal (as expected from the phylogeny in Fig 2), with a main mode at 4 Single Nucleotide Polymorphisms (SNPs), and the presence of distinct lineages shown by some sequences differing by over 700 SNPs.

**Fig 4:**
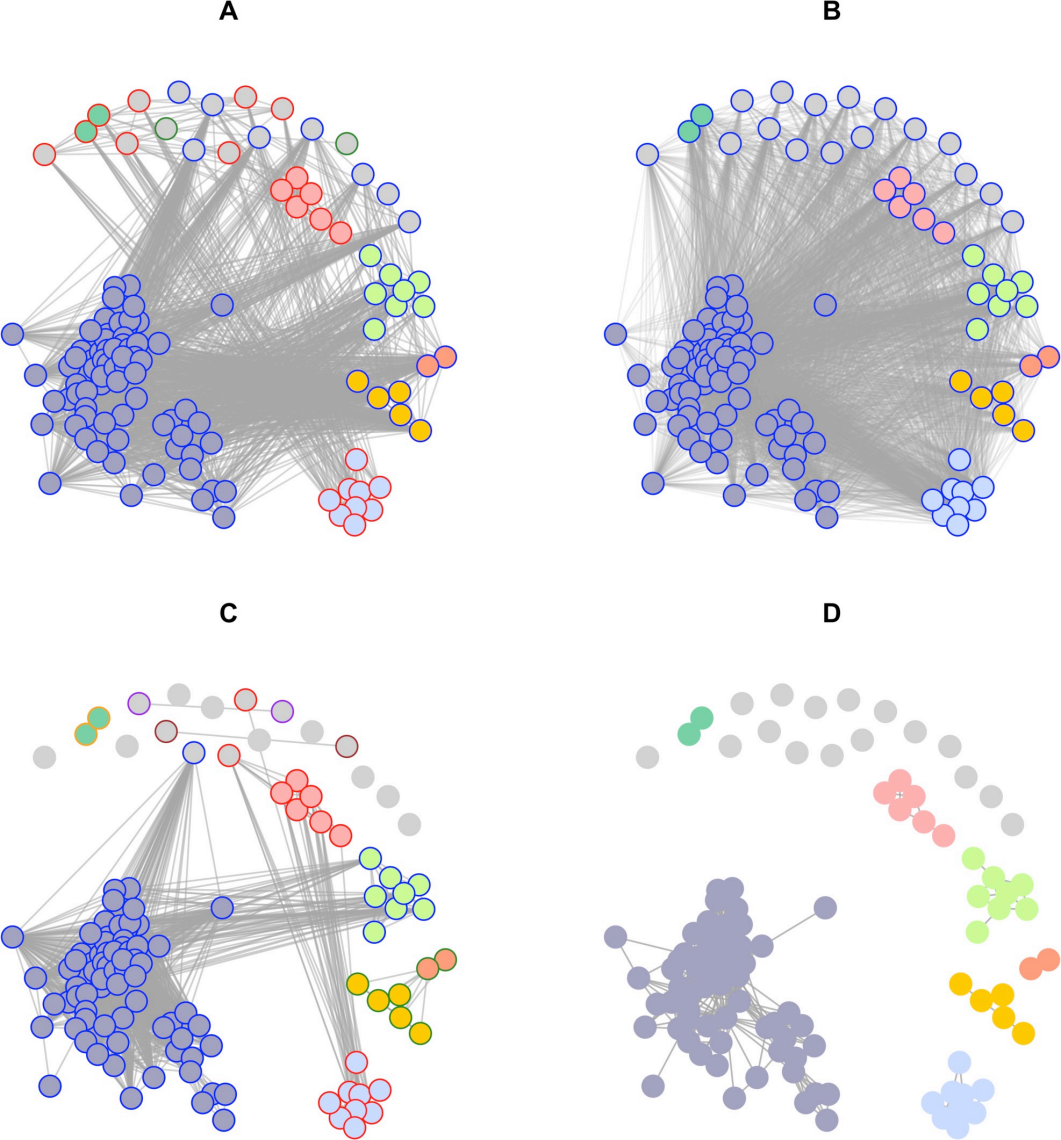
Pruned (A-C) and final graph (D) used to define clusters of cases in the rabies outbreak, obtained using A) only temporal distances, B) only spatial distances, C) only genetic distances and D) all three combined. Nodes represent cases, and edges potential epidemiological links, according to the corresponding data. The inner colours of the nodes indicate the final clusters obtained by combining all data streams (D), whilst the outer colours correspond to the clusters obtained using one data stream at a time. Grey indicates singletons. A reporting rate of 20% was assumed, and pruning cutoff distances corresponding to the 95% quantiles of the input distance distributions were used (see S1 Text for sensitivity analyses on these assumptions). In each graph, the transparency of the vertices was adjusted according to the number of vertices in the graph, with more transparency in graphs with more vertices, to improve readability.

Fig 3 shows, overlaid onto the observed pairwise distances, the distributions for the expected distances (in time, space or genetic) between a case and its closest observed ancestor based on the literature and given an assumed reporting rate of 20% [39]. The equivalent distributions with no underreporting, i.e. the distributions of expected distances between a case of rabies and its infector are shown in S1 Fig. Based on these input distributions, we defined cutoffs for each data stream corresponding to the 95% quantiles (90 and 98% quantiles were also considered in sensitivity analyses, see S1 Text). The observed temporal, geographic, and genetic distances were combined with the input cutoffs to produce pruned graphs for each data stream (Fig 4A–4C). These graphs were in turn intersected to produce the final graph whose connected components are clusters of cases connected in all dimensions, therefore representing cases likely linked by transmission (Fig 4D).

Interestingly, the clusters identified in the final graph (Fig 4D) could not be revealed by any data stream alone (Fig 4A–4C), which illustrates the value of integrating all data streams to identify outbreak clusters. The temporal data alone identified only three clusters of size 2, 23 and 98 respectively; the spatial data could not distinguish any separate introductions; finally, the genetic data led to 9 singletons, three pairs, and three larger clusters of size 7, 17 and 84 respectively. Using all data streams together, a total of 23 distinct clusters were identified, including 16 singletons, two pairs and 5 larger clusters containing respectively 5, 6, 8, 9, and 75 observed cases. These clusters are overlaid on the original data in Fig 2. Importantly, the fact that spatial data alone did not identify any cluster does not mean that this data contained no information on potential clusters. In fact, spatial data was useful in combination with temporal and genetic information, allowing to disentangle outbreak clusters which were otherwise seen as a single outbreak when considering temporal and genetic data only.

For a given reporting rate, the size of outbreak clusters contains information on transmissibility, as measured by the reproduction number (*R*). Intuitively, low values of R will result in many sporadic cases, imported from outside of the reporting area but without subsequent transmission, while higher values of *R* would result in a smaller number of larger clusters. Using the distribution of the cluster sizes (Fig 4D), we were able to estimate *R* as well as the number of unobserved outbreak clusters, which we translated into a rate of importation of rabies into the population over the monitored time period. The estimated reproduction number was 0.92 (95% confidence interval CI_95%_: [0.85; 1.01]) and the estimated rate of importation of rabies into the population was 4.91 (CI_95%_: [3.60; 6.65]) importations per year (see Table 1).

**Table 1:** Estimates of the reproduction number (*R*) and rate of importation of rabies into the canine population (total and unobserved). The rate of importation was defined as the estimated number of outbreaks per unit of time over the whole study period. The rate of unobserved importation was defined as the estimated number of unobserved outbreaks per unit of time over the whole study period. A reporting rate of 20% was assumed, and pruning cutoff distances corresponding to the 95% quantiles of the input distance distributions were used (see S1 Text for sensitivity analyses on these assumptions).

Parameter	Central estimate	95% Confidence interval
Reproduction number (R)	0.92	0.85–1.01
Yearly rate of importation	4.91	3.60–6.65
Yearly rate of unobserved importation	2.40	1.09–4.14

The results presented above were obtained by assuming a reporting rate of 20%, and using a cutoff corresponding to the 95% quantile of the input distance distributions at the pruning step. We explored the extent to which results were affected by changes in these assumptions in sensitivity analyses presented in S1 Text, where reporting rates of 10%, 20% and 50%, and cutoffs corresponding to the 90%, 95% and 98% quantiles were considered. Lower reporting rates and higher quantiles generally led to fewer, larger clusters (S3–S6 Figs). The number of clusters derived varied from 8 to 89 across the scenarios considered. The largest cluster comprised from 21 to 90 cases across scenarios. In all scenarios, the largest cluster included cases occurring in 2007 when a surge in reported cases was observed. The estimates of the reproduction number in the two most extreme scenarios were 0.41 (CI_95%_: [0.31; 0.52] for a reporting rate of 50% and a cutoff corresponding to the 90% quantile) and 0.98 (CI_95%_: [0.93; 1.04] for a reporting rate of 10% and a cutoff corresponding to the 98% quantile) respectively. The estimated rate of importation of rabies into the population for these two extreme scenarios was 15.70 [95%CI 13.74; 18.10] and 2.07 [95%CI 1.20; 3.60] importations per year respectively. More detailed results of these sensitivity analyses are presented in S1 Text.

We have illustrated our method by applying it retrospectively to the data on dog rabies in Central African Republic. However, our method would also be extremely useful in real-time for disentangling clusters of related cases from isolated cases due to separate introductions of the pathogen into the population. Indeed, this could have a direct impact on control policies, providing information in real time on whether to prioritise control measures aiming at reducing transmission or at reducing importations (or both). In S1 Text, we present a sensitivity analysis where our method is applied to the rabies dataset at two intermediate time points, as well as at the end. We show that our method is robust to application in real-time, with very few differences between the allocation of cases to different clusters in real-time and retrospectively (see S1 Text for detailed results).

**Fig 5:**
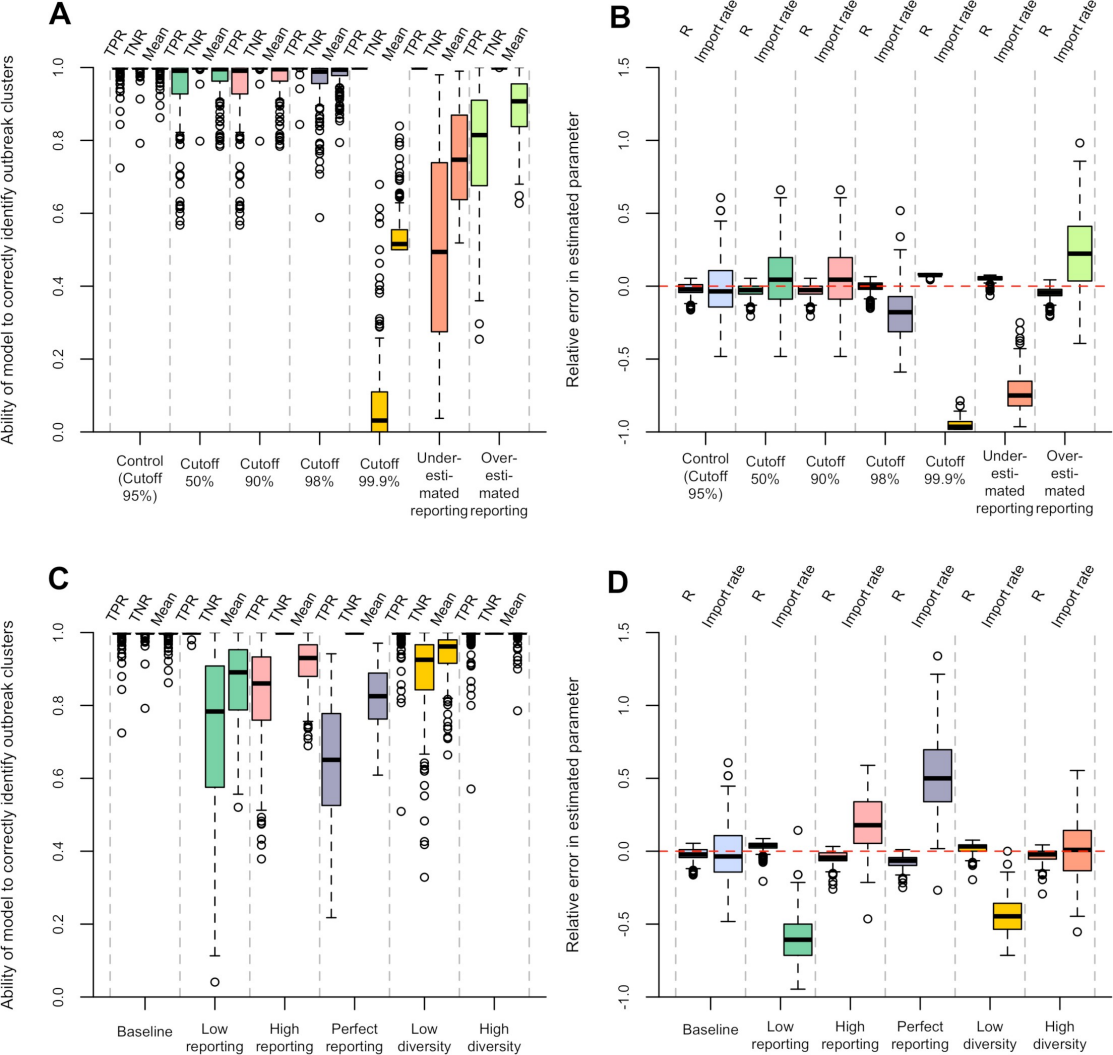
Summary of the simulation study. **A, B**: For the baseline simulation (mimicking rabies transmission in Bangui), performance of the method using different reconstruction scenarios, varying in terms of cutoff used at the pruning step and assumed reporting rate. **C, D**: Performance of the method, using the control reconstruction scenario (i.e. assuming transmission and evolution parameters as well as reporting rate are known), applied to different simulation scenarios varying in terms of reporting rate and diversity of the pathogen in the imported cases. See materials and methods and S1 Text for definition of all the simulation and reconstruction scenarios. Panels **A** and **C** show, across these scenarios, the ability of the model to correctly identify outbreak clusters, as measured by the true positive rate (*TPR*, proportion of pairs of cases belonging to the same transmission tree who are inferred to be in the same outbreak cluster), the true negative rate (*TNR*, proportion of pairs of cases not belonging to the same transmission tree who are assigned to different outbreak clusters), and the mean between *TPR* and *TNR*. Panels **A** and **C** show, across all scenarios, the distribution of the relative error in the estimated reproduction number (*R*) and importation rate.

We also performed a simulation study to assess the extent to which our method was able to correctly identify clusters of cases linked by transmission and accurately estimate the underlying reproduction number and reporting rate. Results are presented in Fig 5. Our method performed generally very well, but the cutoff choice affected the results dramatically. In our main analysis of simulated datasets aiming to reproduce the Bangui epidemiological situation, the mean sensitivity (measured as the True Positive Rate, TPR, see materials and method) was 99.3% with a mean specificity (measured as the True Negative Rate, TNR) of 99.7%. The corresponding median relative error in the estimated parameters was low, at -2.5% for the reproduction number and -0.8% for the importation rate (see S1 Text for more results). These results were obtained assuming the same reporting rate as in the simulation, and with a cutoff corresponding to the 95% quantile of the input distributions. Using higher quantiles generally led to a higher sensitivity, but a lower specificity. The average between sensitivity and specificity was very high for all but the highest cutoff considered (99.9% quantile), and was maximal for the 95% quantile (S12 and S13 Figs). Misspecifying the reporting rate led to lower performances: underestimating and overestimating the reporting rate were associated respectively with lower specificity and lower sensitivity (Fig 5A). In other simulation scenarios with different reporting rates, using the 95% quantile for pruning led to good although slightly poorer performance, with a mean average between sensitivity and specificity greater than 82%. Varying the degree of diversity in the pathogen sequences of the imported cases didn’t affect much the performance of the method (mean average between sensitivity and specificity greater than 93%, Fig 5C).

## Discussion

We have described a new method to identify clusters of related cases of an infectious disease by combining several data streams. Our study used temporal, spatial and genetic data to detect outbreak clusters, but there is no theoretical limit to the number and nature of data sources, as long as the data can be used to compute a measure of dissimilarity between pairs of cases.

Unlike previous approaches [38], our method also exploits preexisting information on the disease, such as its serial interval, spatial kernel, and mutation rate, to define whether cases are part of the same outbreak cluster or not. Our method will therefore be particularly useful to understand transmission patterns for known pathogens, for which the natural history, transmission characteristics and evolutionary rates are well described in the literature. On the other hand, non-parametric approaches such as that described in Ypma et al. [38], which make no assumption about the underlying pathogen, may be better suited to the analysis of data on emerging, poorly characterised pathogens. Note however that the performance of the method described in Ypma et al. [38] has only been tested on data simulated using an extremely simple and unrealistic evolutionary model, with at least 80% reported cases. In contrast, our approach explicitly accounts for underreporting, and was tested on more realistic epidemic scenarios, with reporting rates as low as 10%. This makes our method more widely applicable in real-life epidemic investigations, in which fully observed transmission chains are rare.

We used our approach to analyse a dataset of dog rabies in Central African Republic, previously studied using a combination of methods, including a much more complex approach involving particle Monte Carlo Markov chain inference [39]. The authors estimated an annual rate of 6.78 importations per year ([CI_95%_: [3.65; 14.08]), well in line with our main estimate of 4.91 (CI_95%_: [3.60; 6.65]) and broadly consistent with estimates obtained in our sensitivity analyses (central estimates ranging from 2.07 to 15.70 importations a year depending on the reporting rate assumed and the input cutoff used). Similarly, Bourhy et al. [39] estimated a reproduction number just below the critical value of 1, which is consistent with our estimate of *R* of 0.92 (CI_95%_: [0.85; 1.01]), with central estimates ranging from 0.41 to 0.98 depending on the reporting rate assumed and the input cutoff used). Simply put, our approach retrieved qualitatively identical results to the previous, more complex study, whilst being substantially simpler and orders of magnitude faster.

Our simulation study confirmed that our method runs fast (S14 Fig) and is able to correctly identify clusters of cases linked by transmission, and to subsequently accurately estimate the underlying reproduction number and importation rate. Importantly, our simulations also highlighted the importance of adequately choosing the cutoffs used at the pruning step. In our rabies application, we used cutoffs corresponding to the 95% quantiles, identified in our simulation study as maximising the average between sensitivity and specificity (S12 and S13 Figs). In practice, optimal cutoffs will likely vary across datasets, diseases and epidemic contexts. Different strategies may be employed to address the issue. First, sensitivity analyses should be performed to study the impact of changing the cutoffs and reporting rates, as has been done here. Second, whenever possible, simulations can be used to identify optimal cutoffs for a specific outbreak context. Finally, further work may be dedicated to developing automated frameworks for the selection of optimal cutoffs from past outbreak data in which clusters have been identified through epidemiological investigations.

Interestingly, the identification of optimal cutoffs may not always be necessary. In some cases, one may choose to prioritise high sensitivity or high specificity depending on the relative importance of not detecting an importation versus misclassifying a transmission link as an importation. For example our method could be used with high cutoffs to design a highly sensitive surveillance tool, which would then trigger a warning calling for closer examination of the suspected new outbreak clusters.

Beyond its computational efficiency, our approach is also extensible to most other infectious diseases, and can be used to achieve epidemic evidence synthesis from any data. For instance, contact data can readily be incorporated in the form of binary distances, where the distance between cases with a reported contact is zero, and one otherwise. Our approach could use any measure of social [43–45] or ecological distances [46], as well as measures of dissimilarity based on reported symptoms (e.g. proportion of different symptoms reported by a pair of cases). Whenever deep sequencing data is available, existing measures of the genetic differentiation between populations can also readily be applied to describe genetic distances between individuals whilst accounting for within-host diversity [47–50]. An additional advantage of our approach for data integration is that it does not formally require different data streams to be independent. Rather, independence is a practical condition for the method to be more efficient: ideally, different data sources should be complementary rather than redundant, so that combining them effectively improves the identification of clusters of related cases.

One current limitation of our method relates to partially missing data, i.e. cases documented on some but not all data streams (e.g. cases with an onset date but no location). So far, our method assumes that reported cases are documented for all the data sources. As our method explicitly incorporates underreporting, such partly missing data are currently discarded and the reporting probability adjusted accordingly. However, further work should be dedicated to dealing with partly missing data in a more constructive way.

As in Bourhy et al. [39], we rely on a Poisson model for estimating *R* and the number of unobserved outbreak clusters, which assumes homogeneous infectiousness amongst cases. Although the presence of superspreading would have no impact on the outbreak clusters identified by our approach, it would likely lead to underestimating both *R* and the number of unobserved outbreak clusters [51]. Future research could extend the current methodology to account for such heterogeneity.

The integration of this new approach in existing surveillance systems should be facilitated by its ability to accommodate different distance measures and its implementation in the free, fully documented and open-source package *vimes* (www.repidemicsconsortium.org/vimes) for the R software [52]. Being an evidence synthesis method at core, *vimes* benefits from the wealth of tools for analysing different types of data available in R, including time series analysis [53,54], geographic information systems [GIS, 54], population genetics [47–50,55], phylogenetics [56–58], and infectious disease epidemiology [59–61]. We hope this environment, together with the simplicity and flexibility of the method, will make it an effective and practical contribution to infectious disease surveillance.

## Materials and methods

### Evidence synthesis using a graph approach

#### Rationale

The rationale for our approach is illustrated in Fig 1 with a schematic example including two data streams: geographical and genetic data. For each stream of data, pairwise distances between cases are computed and used to define pruned graphs where only potentially related cases are connected. These graphs are then merged into a single graph whose connected components represent likely outbreak clusters. The distribution of the sizes of these clusters is finally used for estimating transmissibility, as measured by the reproduction number *R*.

We first consider each data stream independently. For each data stream *n* we define a dissimilarity measure *d*, used to compute distances between all pairs of cases (Fig 1A and 1D). We denote *d*^*n*^_*i*,*j*_ the distance between cases *i* and *j* with respect to data stream *n*. We define a weighted graph linking all cases (nodes), where the weight of the edge between two cases *i* and *j* is given by *d*^*n*^_*i*,*j*_ (Fig 1B and 1E). We then prune the graph (Fig 1C and 1F) by removing all edges with weights above a predefined cutoff κ^*n*^, (see two sections below for considerations on cutoff choice). The resulting graph *G*^*n*^ = {*N*, *E*^*n*^} contains all cases (nodes *N*), and connects cases by edges (*E*^*n*^) if and only if the corresponding pairwise distance is less than κ^*n*^, so that *E*^*n*^ = {(*i*,*j*) | *d*^*n*^_*i*,*j*_ ≤ κ^*n*^ ∀ *i*,*j* ∈ *N*}. Simply put, the graph *G*^*n*^ only connects cases which are similar to each other with respect to data stream *n*.

The final step of our approach consists in combining all pruned graphs into a single graph *G* by intersection (Fig 1G), which we formally note *G* = ⋂_*n*_ *G*^*n*^. The graph intersection is achieved by retaining edges present in all the individual graphs, so that *G* = {*N*, *E*} with *E* = {(*i*,*j*) | (*i*,*j*) ∈ *E*^*n*^ ∀ *n*}. Simply put, cases remaining connected in this final graph have to be connected in all data streams, so that the connected components of *G* identify clusters of cases likely to belong to the same outbreak cluster.

#### Data streams and distances

Our method is very generic and could be applied using any number and type of data streams, and any distance metric for a given data stream. However, we explore in detail three data streams for which data is commonly available: data on the timing of infection of cases, data on the location of cases, and WGS of the pathogen sampled from each case. For the temporal data stream, we define the distance between two cases as the delay between a specific clinical event (typically symptoms, but could be anything else) in the two cases. For the spatial data stream, we consider the Euclidean distance between the locations of each pair of cases (e.g. the location of their home but again could be defined differently). Finally, for genetic data, we measure the distance between two cases as the Hamming distance, defined as the number of Single Nucleotide Polymorphisms (SNPs) between the pair of considered sequences.

#### Choice of cutoffs

The choice of the cutoff κ^*n*^ for each data stream will dramatically influence the number of edges retained through the pruning process, and in turn the size of the clusters derived by our method. Here, we consider the case where the pathogen of interest is already known, or where existing case-investigation data exist, so that preexisting information on the distribution of expected distances between cases can be used as input to inform the cutoff choice. If *f*^*n*^ denotes the input probability density function or probability mass function of expected distances between a case and its infector for data stream n, the cutoff κ^*n*^ for that data stream could then be defined as a predetermined quantile of *f*^*n*^, for instance the 95% quantile. With this approach, two cases would be considered disconnected with respect to data stream *n* (after the pruning step), if the distance between them *d*^*n*^_*i*,*j*_ is larger than the 95% quantile of the input distribution *f*^*n*^. Note that, when several independent data sources are combined, the quantile could be chosen to ensure a certain sensitivity. For instance in our application with 3 data streams, a quantile of 95%^1/*3*^ ≈ 98% in each of the *3* data streams would lead to an overall sensitivity of 95%. Note however that this assumes independence between all data streams, which in practice would rarely be the case. For instance, if a secondary case arises soon after a primary case, it is likely to also be close to the primary case in geographical and genetic space. In the presence of such correlations, the resulting sensitivity is likely to be larger than 95%.

#### Underreporting

Typically in an outbreak, reporting is incomplete, for example due to imperfect surveillance systems, asymptomatic cases, or patients not seeking care. Such underreporting will directly affect the distances between observed cases, with more underreporting leading to larger distances. For instance, for the temporal data stream, the delay between symptom onset in a case and its closest observed ancestor is typically longer as underreporting, and hence the number of unobserved intermediate cases, increases. If the overall reporting probability, π, is known, and assuming that the probability of being reported is identical for all cases, the number of unobserved intermediate cases between two observed cases can be described by a geometric distribution with probability π. Using this property, we propose, in that case, to define the cutoff κ^*n*^ as a quantile of *f*^*n*,*π*^, the distribution of the expected distance between an observed case and its closest observed ancestor, accounting for potential unobserved intermediate cases. The formula to derive *f*^*n*,*π*^ is presented and explained in S1 Text. Note that if all cases are observed i.e. π = 100%, then *f*^*n*,*π*^ = *f*^*n*^, as in the previous section.

#### Analytical results in special cases

For the three data streams detailed here, we assume that data is available to inform the distribution of the serial interval (the time between a given event such as symptoms onset in a case and its infector), the spatial kernel (the distribution of geographical distances between a case and its infector), and the mutation rate per genome per generation (which, combined with the distribution of the serial interval, can be translated into a distribution of the number of SNPs between a case and its infector [62]). For the input distributions *f*^*n*^, we consider specific parametric distributions that are particularly appropriate to describe these three data streams and allow us to derive an analytical formulation for *f*^*n*,*π*^, in the presence of underreporting (S1 Text). We use Gamma, Rayleigh, and Poisson distributions for *f*^*n*^ for temporal, spatial and genetic distances respectively. For each of these cases, we provide analytical formulas for *f*^*n*,*π*^, the input probability density function or probability mass function of the distance between two cases, accounting for underreporting. These can then be used to derive corresponding quantiles, and hence cutoffs to be used at the pruning step.

#### Availability

The method is implemented and documented in the package *vimes* for the R software [52], available on github (http://github.com/reconhub/vimes) and documented in a dedicated website (http://www.repidemicsconsortium.org/vimes). The analysis of one of our simulations is entirely reproduced in a dedicated vignette, which can be accessed in R by typing the three following commands:

devtools::install_github("reconhub/vimes", build_vignettes = TRUE)

vignette(package = "vimes") # list available vignettes

vignette("rabies", package = "vimes") # open rabies vignette

### Estimation of *R* from the distribution of cluster sizes

Transmissibility of a pathogen can be measured using the reproduction number *R*, defined as the average number of secondary cases infected by a single primary case. Here we assume that the number of secondary cases (or ‘offsprings’) follows a Poisson distribution with mean *R*. Intuitively, the final size (i.e. the total number of cases once extinction has occurred) of an outbreak seeded with one infected individual, is related to *R*. Numerous studies have characterised this relationship and provided formulations to estimate the reproduction number from the final size of naturally extinct outbreaks [8,63]. We built on such methods to account for under-reporting (see S1 Text). Assuming unobserved cases are distributed at random across the outbreaks, this approach adjusts the likelihood of an observed outbreak final size by integrating over all possible unobserved cases in that outbreak. Importantly, this method also accounts for the possibility for some, typically small outbreaks, to go entirely unobserved. The method estimates *R*, as well as the probability of observing an outbreak, *P*_*obs*_. An estimate of the number of unobserved outbreaks may then be obtained assuming that it follows a negative binomial distribution characterised by *P*_*obs*_ and the number of observed outbreaks. A full description of the inference on both the reproduction number and the number of importations can be found in S1 Text. The method is implemented and documented in the package *branchr* for the R software [52], available on github (http://github.com/reconhub/branchr).

In summary, given a set of observed outbreak cluster sizes, we obtain a maximum likelihood estimate of *R* (and associated confidence intervals), as well as estimates of the total number of imported cases accounting for under-reporting. A rate of importation is then derived from the total number of imported cases and the period covered by the surveillance system.

### Analysis of the rabies data

#### Data

The Institut Pasteur of Bangui, under the instruction of the State health department of the Central African Republic, conducts the collection of suspected rabies dogs and performs rabies laboratory diagnosis according to established national standardized protocols and WHO guidelines [64,65]. Over the period 2003–2012, a linelist of 151 rabies cases was collated, with information on the date of report, the spatial coordinates (for all the isolates originating from Bangui), and the genetic sequence of the isolated virus. Rabies diagnosis was performed by direct immunofluorescence on smears of suspected animals, and confirmed in parallel using RT-PCR [64,66]. Viral RNA from brain samples was extracted using Trizol reagent (Invitrogen) according to the manufacturer’s instructions. RNA extractions, RT-PCR and sequencing were performed as described previously [39]. 42% of the viral genome was sequenced, including the N, P, M, G and intergenic G-L region, corresponding to positions 161 to 5,221 of the RABV genome [67]. All 151 sequences were aligned using the MUSCLE [68] plugin for Geneious 5.6.6 (Biomatters).

The dataset is available in the R package ‘outbreaks’, and can be accessed in R by typing the three following commands:

devtools::install_github("reconhub/outbreaks")

library(outbreaks)

?rabies_car_2003

#### Parameters

Information on the distributions of expected distances between rabies cases was retrieved from the literature to inform the cutoff choice used at the pruning step. Following Hampson et al. [69], we assumed a Gamma distributed serial interval, with mean 23.6 days and standard deviation 20.9 days [69]. We assumed a Rayleigh distributed spatial Kernel with scale 0.70km, consistent with a mean transmission distance of 0.88 km, as in Hampson et al. [69] (see S1 Text). We assumed a substitution rate of 5.9 x 10^−4^ substitutions per site per year for rabies, as estimated in Bourhy et al. [39]. The distributions of the serial interval, spatial kernel, and number of mutations between transmission pairs are shown in S1 Fig. We considered, as in Bourhy et al. [39], a reporting rate of 20% in our main analyses (presented in the main text), and reporting rates of 10 and 50% respectively in two extreme scenarios considered in sensitivity analyses (shown in S1 Text). Finally, in our main analyses we used the 95% quantile of all input distributions as cutoff at the pruning step. Cutoffs corresponding to the 90% and the 98% quantiles were also considered in sensitivity analyses (S1 Text).

### Simulations

We performed a simulation study to assess the ability of our method to correctly identify clusters of cases linked by transmission and to accurately estimate the underlying reproduction number and importation rate. We considered six simulation scenarios mimicking the transmission of rabies among dogs. Our baseline scenario was designed to closely mirror the transmission characteristics underlying the transmission of rabies in our dataset from Bangui. We then considered five variations of this scenario: a ‘low’, ‘high’ and ‘perfect’ reporting scenarios similar to the baseline scenario but with varying levels of reporting, and a ‘low’ and ‘high’ diversity scenarios, similar to our baseline but where the imported cases had pathogen genetic sequences respectively much more similar and much more different to one another. The scenarios are described in S1 Table.

For each simulation scenario, we used our method to reconstruct the clusters of cases linked by transmission, as well as re-estimate the reproduction number and the importation rate. In the reconstruction process, we used input distributions obtained by assuming the same mutation rate, genome length, serial interval and spatial kernel as in the simulation. For the analysis of the baseline simulations, we systematically varied the cutoffs and reporting rate, as described in S2 Table.

Detailed information on the simulation process is available in S1 Text.

Following Beugin *et al*. [35], we quantify the ability of our method to correctly identify clusters of cases linked by transmission, through measuring 1) the true positive rate (*TPR*) or sensitivity, i.e. the proportion of pairs of cases belonging to the same transmission tree who are inferred to be in the same outbreak cluster), 2) the true negative rate (*TNR*) or specificity, i.e. the proportion of pairs of cases not belonging to the same transmission tree who are assigned to different outbreak clusters and 3) the mean between *TPR* and *TNR*, which is proportional to the Rand index, a common criterion used to evaluate clustering methods [35,70]. We also compare the estimates of the reproduction number and importation rate to the values used in the simulation.

## Supporting information

S1 Text Additional methods and results. (PDF)

S1 Fig Distributions of distances assumed for rabies and used to determine the cutoffs to be used at the pruning step. Top: Distribution of the serial interval in days (here defined as the time between reporting of a case and its infector). Middle: Distribution of the spatial kernel in kilometers (the distance between a case and its infector). Bottom: Distribution of the number of mutations between the whole genome sequence of the pathogen sampled from a case and its infector.
(PDF)

S2 Fig Distribution of pairwise temporal (left), spatial (middle) and genetic (right) distances for rabies. The temporal distance is defined as the time between sampling of the pathogen in cases. The spatial distance is defined as the Euclidean distance between the locations of cases. The genetic distance is defined as the number of Single Nucleotide Polymorphisms (SNPs) between the Whole Genome Sequences (WGS) sampled from cases.
The grey histograms show the observed pairwise distances between any two cases reported in the Bangui outbreak. The solid black lines show the assumed distribution of distances between a case and its closest observed ancestry, given an assumed reporting rate of 10% (top row), 20% (middle row) and 50% (bottom row); note that the distributions have been rescaled to fit on the same graph as the histograms. The vertical lines show the cutoffs corresponding to the 90% (yellow), 95% (orange) and 98% (red) quantiles of these distributions.
For a given data stream and a given choice of reporting rate and cutoff, pairs of cases with observed distance above the cutoff are considered not connected, and the corresponding graph edges are removed at the pruning step (see Fig 1 in main text).
(PDF)

S3 Fig Graph representation of clusters of cases in the rabies outbreak, obtained from temporal, spatial and genetic data together, and using different assumptions on the reporting rate (10%, top row; 20%, middle row; and 50%, bottom row) and cutoff for pruning (corresponding to the 90% quantile, left column; 95% quantile, middle column; and 98% quantile; right column). The colours of the nodes (i.e. cases) correspond to the cluster they belong to according to the analysis using a given combination of reporting rate and cutoff. Grey indicates singletons.
(PDF)

S4 Fig Visualization of the clusters of cases of rabies in time, using different assumptions on the reporting rate (10%, top row; 20%, middle row; and 50%, bottom row) and cutoff for pruning (corresponding to the 90% quantile, left column; 95% quantile, middle column; and 98% quantile; right column). The colours correspond to the cluster cases are inferred as belonging to according to the analysis using a given combination of reporting rate and cutoff. Grey indicates singletons.
(PDF)

S5 Fig Visualization of the clusters of cases of rabies in space, using different assumptions on the reporting rate (10%, top row; 20%, middle row; and 50%, bottom row) and cutoff for pruning (corresponding to the 90% quantile, left column; 95% quantile, middle column; and 98% quantile; right column). The colours correspond to the cluster cases are inferred as belonging to according to the analysis using a given combination of reporting rate and cutoff. Grey indicates singletons.
(PNG)

S6 Fig Visualization of the clusters of cases of rabies on phylogenetic trees. The two clades referred to as A (top) and B (bottom) in the main text are represented separately for more readability (clades are distant by more than 700 nucleotides, masking all other diversity). The full phylogeny was obtained by Neighbour-Joining on Hamming distances. Results are shown for various combinations of the reporting rate (10%, top row; 20%, middle row; and 50%, bottom row) and cutoff for pruning (corresponding to the 90% quantile, left column; 95% quantile, middle column; and 98% quantile; right column). The colours correspond to the cluster cases are inferred as belonging to according to the analysis using a given combination of reporting rate and cutoff. Grey indicates singletons.
(PDF)

S7 Fig Estimated reproduction number (top) and yearly rate of importation of rabies in the population (bottom), for different assumptions on the reporting rate (10%, 20% or 50%) and cutoff for pruning (corresponding to the 90%, 95% or 98% quantile). The dots correspond to the maximum likelihood estimates and the vertical bars show the 95% confidence intervals around these.
(PDF)

S8 Fig Visualization of the clusters of cases of rabies in time, inferred at different times in the outbreak (top: 24 April 2007; middle: 06 June 2008; bottom: 06 March 2012), assuming a reporting rate of 20% and using a cutoff for pruning corresponding to the 95% quantile. The colours correspond to the cluster cases are inferred as belonging to according to the analysis performed at each time point. Grey indicates singletons. The vertical lines show the three dates at which the algorithm was run.
(PDF)

S9 Fig Distribution of the simulated dataset sizes, for each of the six simulation scenarios considered (see S1 Text and S1 Table for a definition of the simulation scenarios). (PDF)

S10 Fig Reestimated reproduction number (left) and importation rate (right), for each of the seven reconstruction scenarios applied to the 'baseline' simulation scenario (top) and for the 'control' reconstruction scenario applied to each of the six simulation scenarios (bottom). See S1 Text, S1 Table and S2 Table for a definition of the simulation and reconstruction scenarios. The dots show the central estimates for each simulation; the vertical bars show the 95% confidence intervals. The red horizontal dashed lines show the values used in the simulation. Note in the perfect reporting scenario, there is no uncertainty on the estimate of the importation rate.
(PDF)

S11 Fig Root mean square error in the estimated reproduction number (R, left axis) and in the estimated importation rate (right axis), for each of the seven reconstruction scenarios considered (see S1 Text and S2 Table for a definition of the reconstruction scenarios).
(PDF)

S12 Fig Distribution of the optimal cutoff choice (defined as that yielding the highest average between sensitivity and specificity), for the 200 baseline simulations. Note that for certain simulations, several cutoffs yielded the same performance, so a single simulation may be contributing to several cutoffs.
(PDF)

S13 Fig ROC curve obtained by applying our method to one simulated dataset, using cutoffs corresponding to the 50%, 75%, 90%, 95%, 95%^1/3^ = 98.3%, 99%, 99.9%, 99.95%, and 99.99% quantiles of the input distributions respectively. The simulated dataset was selected, among the 200 baseline simulations, as the only simulated dataset which happened to have the same size as our rabies dataset, i.e. 151 observed cases. TPR: True Positive Rate; TNR: True Negative Rate.
(PNG)

S14 Fig Vimes computing time, for all simulation scenarios, under the "control" reconstruction scenario (see S1 Text, S1 Table and S2 Table for a definition of the simulation and reconstruction scenarios). The computing time encompasses the time required to 1) compute the pairwise distances in time, geographical space, and genetic space, 2) calculate the cutoffs associated with the quantile chosen (here 95%) and the assumed reporting rate (here 20%), and 3) construct, prune, and merge the graphs to define clusters of cases linked by local transmission. Note the computing time only measures the time required to perform the reconstruction, not the simulation. The dashed line represents y = 2.33 + 1.37 * 10^−5^ x^2.2^.
(PDF)

S1 Table Simulation scenarios. MRCA: most recent common ancestor; -: as baseline; sd: standard deviation; *: https://www.ncbi.nlm.nih.gov/nuccore/JQ685977.1
(PDF)

S2 Table Reconstruction scenarios. (PDF)

S1 Code R code used to perform the simulations. (R)
