# Supplementary material for: A graph-based evidence synthesis approach to detecting outbreak clusters: An application to dog rabies
Source: PLoS Comput Biol. 2018 Dec 17;14(12):e1006554. doi: 10.1371/journal.pcbi.1006554 (PMC6312344; doi:10.1371/journal.pcbi.1006554)
Supplement: S1 Text — (PDF) [file pcbi.1006554.s001.pdf]

Supplement for: A graph-based evidence synthesis approach to detecting outbreak clusters: an application to dog rabies

Anne Cori, Pierre Nouvellet, Tini Garske, Hervé Bourhy, Emmanuel Nakouné, Thibaut Jombart

Contents

Overview **2**

Choosing the cutoffs based on preexisting information about the pathogen of interest **2**

Notations . . . 2
Distribution of the distance between two cases accounting for underreporting . . . 3
Special case 1: ϕ is the pdf of a Gamma distribution (typical for serial interval distribution) . . . 3
Special case 2: ϕ is the pmf of a negative binomial distribution (typical for transmission divergence distribution) . . . 3
Special case 3: ϕ is the pdf of a Rayleigh distribution (typical for spatial kernel distribution) . . . 4

Estimation of the reproduction number **5**

General approach . . . 5
To estimate the reproduction number . . . 5
To estimate the number of importations . . . 6
Practical considerations . . . 6
To estimate the reproduction number . . . 6
To estimate the number of importations . . . 7
Implementation . . . 7

Parameterisation for rabies **7**

Sensitivity analyses **7**

Reporting rate and cutoff choice . . . 7
Real-time application . . . 8

Simulations **9**

Simulation and reconstruction scenarios . . . 9
Simulation process . . . 9
Simulated datasets . . . 9
Reconstruction results . . . 10
Comparing different cutoffs . . . 10
Computing time . . . 10

Overview

In our study, we propose a new method for combining several data streams, e.g. temporal, spatial and genetic data, to identify clusters of related cases of an infectious disease.

A key step in our method consists in defining, for each data stream, a cutoff distance above which cases are considered as not being part of the same outbreak cluster, i.e. not linked by local transmission. The section on 'Choosing the cutoffs based on preexisting information about the pathogen of interest' provides a theoretical background for how to choose the cutoffs.

Once each observed case has been allocated to an outbreak cluster, we propose to use the distribution of cluster sizes to estimate the underlying reproduction number (R, the average number of secondary cases infected by an infected individual). The section on 'Estimation of the reproduction number' describes the method used to estimate R.

We applied our method to analyse dog rabies epidemics data collected in Central African Republic. The section entitled 'Parameterisation for rabies' describes how we parameterised the model for rabies; in particular how we informed the choice of cutoff based on prior information on the serial interval, the spatial kernel and the mutation rate of rabies.

To quantify the impact of the assumed reporting rate as well as the cutoff choice on our results, and to assess the ability of our method to identify outbreak clusters in real-time, we performed sensitivity analyses which are presented in the 'Sensitivity analyses' section.

A more systematic assessment of the performance of our method was conducted through a simulation study, which is presented in the 'Simulations' section.

Choosing the cutoffs based on preexisting information about the pathogen of interest

We consider the case where the pathogen of interest is already known, or where existing case-investigation data exist, so that preexisting information on the distribution of expected distances between cases infected by this pathogen can be used as input to inform the cutoff choice. If f^n denotes the input probability density function or probability mass function of expected distances between a case and its infector for data stream n, the cutoff κ^n for that data stream could then be defined as a predetermined quantile of f^n. Underreporting will directly affect the distances between observed cases, with more underreporting leading to larger distances.

If the overall reporting probability, π, is known, and assuming that the probability of being reported is identical for each case, the number of unobserved intermediate cases between two observed cases can be described by a geometric distribution with probability π. Using this property, we propose, in that case, to define the cutoff κ^n as a quantile of $f^{n,\pi}$, the distribution of the expected distance between an observed case and its closest observed ancestry, accounting for potential unobserved intermediate cases.

In this section, we derive the formula for the input distribution of distances f between a case and its closest observed ancestry in a given data stream, given a certain level of under-reporting. After presenting the general formula, we propose three special cases (i.e. parametric distributions of the distance between a case and its closest observed ancestry) for which the formula simplifies greatly, and which are particularly adapted to describe distances between cases in time, geographical space, and genetic space respectively.

Notations

In all the following, superscripts t, s and g are used to refer to time, geographical space, and genetic space respectively. 'Pdf' stands for probability density function and 'pmf' for probability mass function. For both, we use the same general notation f.

We denote i and j two cases suspected to be linked by a transmission chain, with i being ancestral to j, so that i may be the infector of j, or the infector of its infector, etc.

We denote $\kappa_{i,j}$ the (unobserved) number of generations between i and j, so that $\kappa_{i,j} = 1$ if i is the infector of j, $\kappa_{i,j} = 2$ if i is the infector of the infector of j, etc. We denote π the probability for each infected individual to be reported (assumed equal across all infected individuals).

We denote $d_{i,j}$ the (observed) distance between cases i and j in a given data stream, and ϕ the probability density function of the distance (in time, geographical space or genetic space) between an infected individual and their infector. This can be the serial interval, the spatial kernel, or the transmission divergence (defined, as in Campbell et al. [1] as the number of single nucleotide polymorphisms (SNPs) between the pathogen in a case and in their infector).

In all the following, we often omit the subscripts i, j referring to cases i and j, and we use $\kappa = \kappa_{i,j}$ and $d = d_{i,j}$ for easier reading.

Distribution of the distance between two cases accounting for underreporting

We are interested in computing the probability density function $f(d)$, which can be decomposed according to the unobserved number of missing generations between i and j as follows:

$$f(d) = \sum_{k=1}^{+\infty} f(d|\kappa = k) f(\kappa = k)$$

The first factor in the sum is $f(d|\kappa = k) = \phi^{(k)}(d)$, where $\phi^{(k)}$ denotes the convolution of ϕ with itself, k times ($\phi^{(1)} = \phi$). Note this is assuming that the distance d is additive, so that if i infected l who infected j, then $d_{i,j} = d_{i,l} + d_{l,j}$. This is the case for time, and we assume it is the same for genetic distance. For spatial distance we perform a slightly more complicated reasoning (see below).

The second factor in the sum is the probability of $k-1$ intermediate cases having been unobserved, and the kth case (going back in time, that is i) having been observed. Assuming all infected individual have the same probability π of being reported, this is given by the geometric distribution $f(\kappa = k) = \pi (1-\pi)^{k-1}$.

Therefore:

$$f(d) = \sum_{k=1}^{+\infty} \phi^{(k)}(d) \pi (1-\pi)^{k-1}$$

In the next three paragraphs, we explore special cases where the equation above simplifies greatly, and which are particularly adapted to describe distances between cases in time, geographical space, and genetic space respectively.

Special case 1: ϕ is the pdf of a Gamma distribution (typical for serial interval distribution)

In this section we consider the special case where ϕ is the pdf of a Gamma distribution with shape α and scale β: $\phi(x) = \frac{1}{\Gamma(\alpha)\beta^\alpha} x^{\alpha-1} e^{-x/\beta}$. The sum of k independent variables with same Gamma distribution with parameters (α, β) also has a Gamma distribution, with parameters $(k\alpha, \beta)$. Therefore:

$$f(d) = \sum_{k=1}^{+\infty} \frac{1}{\Gamma(k\alpha)\beta^{k\alpha}} d^{k\alpha-1} e^{-d/\beta} \pi (1-\pi)^{k-1}$$

Special case 2: ϕ is the pmf of a negative binomial distribution (typical for transmission divergence distribution)

The transmission divergence, the expected number of SNPs between two cases, will typically depend on the serial interval (amount of time available to evolve) and the mutation rate. Using a Gamma distribution for the serial interval (with shape α and scale β), and a Poisson distribution for the number of mutations in a given time interval dt (with mean $\mu * dt$) leads to a negative binomial distribution with parameters $\left(\alpha, \frac{\beta\mu}{\beta\mu+1}\right)$ for the number of mutations between two cases. Indeed, if i infected j and $d^g_{i,j}$ is the genetic distance (defined as number of SNPs) between i and j, ϕ_t the (Gamma) pdf of the serial interval, and μ the mutation rate, then the pmf of the genetic distance between i and j is:

$$
\begin{aligned}
\phi^g(d^g) = f(d^g|\kappa=1) &= \int_{t=0}^{+\infty} f\left(d^g|\kappa=1, d^t=t\right)\phi^t(t)\,dt \\
&= \int_{t=0}^{+\infty} \frac{(\mu t)^{d^g} e^{-\mu t}}{d^g!} \frac{1}{\Gamma(\alpha)\beta^\alpha} t^{\alpha-1} e^{-t/\beta} dt \\
&= \frac{\Gamma(\alpha+d^g)\mu^{d^g}}{(\mu+1/\beta)^{\alpha+d^g} d^g!\Gamma(\alpha)\beta^\alpha} \int_{t=0}^{+\infty} \frac{t^{\alpha+d^g-1} e^{-t(\mu+1/\beta)}}{\Gamma(\alpha+d^g)\left(\frac{1}{\mu+1/\beta}\right)^{\alpha+d^g}} dt \\
&= \frac{\Gamma(\alpha+d^g)}{d^g!\Gamma(\alpha)} \frac{\mu^{d^g}}{(\mu+1/\beta)^{\alpha+d^g}\beta^\alpha} \\
&= \binom{\alpha+d^g-1}{d^g} \left(\frac{\beta\mu}{\beta\mu+1}\right)^{d^g} \left(1-\frac{\beta\mu}{\beta\mu+1}\right)^{\alpha}
\end{aligned}
$$

which is indeed the pmf of a negative binomial distribution with parameters $\left(\alpha, \frac{\beta\mu}{\beta\mu+1}\right)$.

Now, the sum of k independent variables with same Negative Binomial distribution with parameters (r,p) also has a Negative Binomial distribution, with parameters (kr,p). Therefore:

$$
\begin{aligned}
f(d^g) &= \sum_{k=1}^{+\infty} \phi^{g(k)}(d^g)\,\pi(1-\pi)^{k-1} \\
&= \sum_{k=1}^{+\infty} \binom{k\alpha+d^g-1}{d^g} \left(\frac{\beta\mu}{\beta\mu+1}\right)^{d^g} \left(\frac{1}{\beta\mu+1}\right)^{k\alpha} \pi(1-\pi)^{k-1}
\end{aligned}
$$

Special case 3: ϕ is the pdf of a Rayleigh distribution (typical for spatial kernel distribution)

We assume that the geographical location of an individual i is given by coordinates (x_i, y_i) in an orthonormal system. We assume that the coordinates of an individual j infected by i are so that $x_j - x_i$ and $y_j - y_i$ are independent and identically distributed according to a centered normal distribution $\mathcal{N}\left(0, \sigma^2\right)$:

$$
f(x_j - x_i|\kappa_{i,j} = 1) = \frac{1}{\sqrt{2\pi\sigma^2}} e^{-\frac{(x_j-x_i)^2}{2\sigma^2}}
$$

Now if i infected an unobserved case l who infected j, then $x_j - x_i = x_j - x_l + x_l - x_i$ so the distribution of $x_j - x_i$ is that of the sum of two independent identical normal variables. This is true as well for $y_j - y_i$, and easily extends to more than 1 unobserved intermediate case. Now, the sum of k independent univariate Normally distributed variables with same mean ν and same variance σ^2 is a univariate Normally distributed variable with mean $k\nu$ and variance $k\sigma^2$ (see `http://www.tina-vision.net/docs/memos/2003-003.pdf` for a proof for $k = 2$). Therefore,

$$
f(x_j - x_i|\kappa_{i,j} = k) = \frac{1}{\sqrt{2\pi k\sigma^2}} e^{-\frac{(x_j-x_i)^2}{2k\sigma^2}}
$$

Using the change of variable technique, one can show that the pdf of $s = (x_j - x_i)^2$, conditional on $\kappa_{i,j} = k$ is $\frac{1}{\sqrt{2\pi k\sigma^2}} e^{-\frac{s}{2k\sigma^2}} \frac{1}{2\sqrt{s}}$. Therefore, conditional on $\kappa_{i,j} = k$, $(x_j - x_i)^2$ follows a Gamma distribution with shape $\frac{1}{2}$ and scale $2k\sigma^2$.

The same reasoning holds for $(y_j - y_i)^2$.

The square of the Euclidean distance between individuals i and j can be computed as ${d^s_{i,j}}^2 = (x_j - x_i)^2 + (y_j - y_i)^2$. Conditional on $\kappa_{i,j} = k$, this is the sum of the squares of 2 independent Gamma distributed

variables with same shape $\frac{1}{2}$ and scale $2k\sigma^2$. Therefore, conditional on $\kappa_{i,j} = k$, ${d^s_{i,j}}^2$ is also Gamma distributed with shape $2 \times \frac{1}{2} = 1$ and scale $2k\sigma^2$, i.e. it is Exponentially distributed with rate $\frac{1}{2k\sigma^2}$.

This means that conditional on $\kappa_{i,j} = k$, the Euclidean distance between individuals i and j, $d^s_{i,j}$ follows a Rayleigh distribution with scale $\sigma\sqrt{k}$ (see general proof here `http://www.math.wm.edu/~leemis/chart/UDR/PDFs/ExponentialRayleigh.pdf`).

Finally,

$$\begin{aligned} f\left(d^s\right) &= \sum_{k=1}^{+\infty} \phi^{s(k)}\left(d^s\right)\pi\left(1-\pi\right)^{k-1} \\ &= \sum_{k=1}^{+\infty} \frac{d^s}{k\sigma^2} e^{-\frac{{d^s}^2}{2k\sigma^2}} \pi\left(1-\pi\right)^{k-1} \end{aligned}$$

Estimation of the reproduction number

In this section we derive key results to estimate the reproduction number and the rate of importation from the number of observed clusters (or outbreaks) and their respective sizes. In the following we assume that the composition of clusters, and most importantly their respective sizes, is known. Therefore, the analysis outlined below does not account for uncertainty in clusters sizes.

General approach

To estimate the reproduction number

Here, we are interested in determining the reproduction number, R, for a given observed outbreak size (assumed to be initiated from a single importation). Following Farrington et al. [2], and assuming a Poisson offspring distribution, we define the probability of an outbreak of size Z occurring given a reproduction number $R < 1$ as:

$$h\left(z; R\right) = Pr\left(Z = z | R\right) = \frac{z^{z-2} R^{z-1} e^{-zR}}{(z-1)!}.$$

However, due to underreporting, only Y cases are observed, with $Y \leq Z$. Denoting π the reporting rate (i.e. the probability for each case to be observed), the probability of observing Y cases from an outbreak characterised by Z cases follows a binomial distribution with parameters Z and π:

$$g\left(y; z, \pi\right) = Pr\left(Y = y | Z = z, \pi\right) \sim Bin\left(Z, \pi\right).$$

Therefore, the probability of observing Y cases given π and R follows:

$$Pr\left(Y = y | R\right) = \sum_{z=y}^{+\infty} Pr\left(Y = y | Z = z, \pi\right) Pr\left(Z = z | R\right) = \sum_{z=y}^{+\infty} g\left(y; z, \pi\right) h\left(z; R\right).$$

For $R > 1$, Waxman et al. (2018) demonstrated that $h\left(z; R\right)$ becomes $h\left(z; Re^{-\alpha}\right) e^{-\alpha}$, with $\alpha = R\left(1 - Re^{-\alpha}\right)$ [3]. Using this correction, inference is not limited to subcritical reproduction numbers.

Given a set of $\{Y_i\}_{i=1,\dots,n}$ observed outbreak sizes, with n the number of observed outbreaks, the likelihood of $\{Y_i\}$ given R becomes:

$$\mathcal{L}\left(R; \{Y_i\}\right) = Pr\left(\{Y_i\} | R\right) = \Pi_{i=1}^{n} \left[\sum_{z=y_i}^{+\infty} g\left(y_i; z, \pi\right) h\left(z; R\right)\right].$$

We must then account for unobserved outbreaks. All of the above assumes that we observe the size of every outbreak. However, an outbreak for which no cases are observed will be missed in our sample, and

thus we need to correct for this form of censoring. Therefore, we normalise the probability of observing Y cases by the probability of observing an outbreak:

$$Pr\left(Y|R,\pi,Y>0\right)=\frac{Pr\left(Y|R,\pi\right)}{1-Pr\left(Y=0|R,\pi\right)}.$$

Having observed n outbreaks of sizes $\{Y_i\}$, the likelihood of $\{Y_i\}$ given R becomes:

$$\mathcal{L}\left(R;\{Y_i\}\right)=Pr\left(\{Y_i\}|R\right)=\prod_{i=1}^{n}\left[\frac{1}{1-\sum_{z=0}^{+\infty}g\left(0;z,\pi\right)h\left(z;R\right)}\sum_{z=y_i}^{+\infty}g\left(y_i;z,\pi\right)h\left(z;R\right)\right].$$

To estimate the number of importations

In a second step, we estimate the number of importations. The total number of importations, N^{imp}, can be split between the number of observed importations, $N^{imp}_{obs}=n$, and the number of unobserved importations, N^{imp}_{unobs}. While the number of observed importations is known (it is the number of distinct outbreaks or clusters observed), the number of unobserved importations needs to be estimated. As seen above, given a reproduction number R, the probability of observing an outbreak is:

$$P_{obs}=Pr\left(Y>0|R,\pi\right)=1-\sum_{z=0}^{+\infty}g\left(0;z,\pi\right)h\left(z;R\right).$$

Therefore, conditional on the reproduction number R and the reporting rate π, the distribution of the number of unobserved importations can be estimated assuming that the number of unobserved outbreaks follows a Negative Binomial with parameters n, P_{obs}.

Hence, it is straightforward to estimate the number of unobserved importations at the reproduction number's MLE. However, to obtain a full estimate of the number of unobserved importations, and in particular to obtain a 95%CI, one needs to integrate over all possible values of R.

Therefore the likelihood of N^{imp}_{unobs} given $\{Y_i\}$ and π can be defined as:

$$Pr\left(N^{imp}_{unobs}|\{Y_i\},\pi\right)=\int_{R=0}^{+\infty}Pr\left(N^{imp}_{unobs}|R,\{Y_i\},\pi\right)Pr\left(R|\{Y_i\}\right)dR.$$

The probability $Pr\left(N^{imp}_{unobs}|R,\{Y_i\},\pi\right)$ can be evaluated as it follows a Negative Binomial distribution with parameter n, P_{obs}. Regarding $Pr\left(R|\{Y_i\}\right)$, we assume it is proportional to the likelihood of $\{Y_i\}$ given R as defined above (i.e. $Pr\left(Y_i|R\right)$). Implicitly, in a Bayesian language, this is to say that the posterior distribution of R is proportional to the likelihood assuming a non-informative prior on R.

Practical considerations

Having established the theoretical framework for inference above, we proceed to explain how it was implemented. In particular, we provide practical solutions to deal with the infinte sum when estimating the reproduction number and how to numerically evaluate the integral when estimating the number of importations.

To estimate the reproduction number

First we evaluate the likelihood of $\{Y_i\}$ given R on a grid of R uniformly distributed. The limits of the distribution can be adjusted and, in our analysis, we chose a grid bounded between 0 and 20 with an accuracy of 0.01. Numerically, we evaluate the likelihood of $\{Y_i\}$ given R by setting an upper threshold on Z, defined as θ. The threshold is internally computed based on the largest outbreak size and the reporting rate. Its value is calculated to ensure that, in the calculation of the likelihood of $\{Y_i\}$ given R , $g\left(max\left(\{Y_i\}\right);z,\pi\right)$ is lower than 10^{-4}.

After obtaining the profile likelihood across the range of R values, we extracted the maximum likelihood estimate (MLE) and its 95% confidence interval (CI) using the Likelihood ratio test framework.

To estimate the number of importations

Once again, a likelihood profile is obtained for a total number of importations ranging from the number of outbreaks observed (no unobserved outbreaks) up to θ_{imp} outbreaks. We then extracted the maximum likelihood estimate (MLE) and its 95% confidence interval (CI) using the Likelihood ratio test framework.

In practice, we manually set the threshold for the maximum number of importation, θ_{imp}. The threshold represents the maximum number of unobserved outbreaks for which the likelihood is evaluated. In our analysis, we manually set this at 1,000.

In our baseline scenario where $\pi = 0.2$ (a fifth of the cases are reported) and the total number of observed outbreaks is smaller than 150, there is more than 99.9% probability that the true number of importations was below 1,000.

In alternative scenarios considered, we set $\pi = 0.5$ and $\pi = 0.1$. If $\pi = 0.5$ (half of the cases are reported) and the total number of observed outbreaks is smaller than 450, then there is more than 99.9% probability that the true number of importations was below 1,000. If $\pi = 0.1$ (a tenth of the cases are reported) and the total number of observed outbreaks is smaller than 70, then there is more than 99.9% probability that the true number of importations was below 1,000.

Those are conservative estimates, as they assume that all unobserved outbreaks are of size 1. In reality unobserved outbreaks may be much larger, therefore the probability of observing them would be larger than π. Should the reporting be much lower or the total number of outbreaks much larger, the threshold can be adjusted (i.e. increased in this case).

Implementation

The method to estimate the reproduction number and the number of importations is implemented in the R package `branchr`, available at `https://github.com/reconhub/branchr`

Parameterisation for rabies

As explained in the methods section, we retrieved information on the typical distances (in time, geographical space, and genetic space) between a case of rabies and its infector from the literature.

Following Hampson et al. [4], we assumed a Gamma distributed serial interval, with mean 23.6 days and standard deviation 20.9 days. Note that throughout our study, we refer to the serial interval, traditionally defined as the time from symptom onset of a case and its infector, however the data from the rabies outbreak we analyse consists in dates of report of the infected animals. Furthermore, in their study, Hampson and colleagues generate estimates of the generation interval, i.e. the time from infection of a case and its infector, which is yet slightly different. Although the serial interval and the generation interval may have different distributions, in general they have the same mean and are often taken as synonymous (see Svensson et al. [5] for more detail).

We assumed a Rayleigh distributed spatial Kernel with scale 0.70km, consistent with a mean transmission distance of 0.88km, as in Hampson et al. [4]. We assumed a substitution rate of 5.9×10^{-4} substitutions per site per year for rabies, as estimated in Bourhy et al. [6]. The corresponded distributions for the serial interval, spatial kernel, and number of mutations between an index and a secondary case are shown in S1 Fig.

Sensitivity analyses

Reporting rate and cutoff choice

The analyses presented in the main text assume a reporting rate of 20%, following Bourhy et al. [6]. Here, we present sensitivity analyses assuming two extreme scenarios (based again on Bourhy et al.) with reporting rates of 10 and 50% respectively. Additionally, the analyses presented in the main text were based on using the 95% quantile of all distributions as cutoff at the pruning step. Here, we present results obtained using cutoffs corresponding to the 90% and the $0.95^{1/3} \approx 98\%$ quantiles respectively. S2 Fig shows, overlaid onto the observed pairwise distances between cases of rabies in this outbreak, the input probability density

function for the distances (in time, space or genetic) between a case and its closest observed ancestry, given an assumed reporting rate of 10, 20, or 50%. Cutoffs corresponding to the 90, 95 and 98% quantiles of these distributions are shown on S2 Fig. Nine combinations of reporting rates (10, 20 or 50%) and quantiles (90, 95 or 98%) were considered in sensitivity analyses. Each analysis differed from the others in that, at the pruning step of our algorithm (see methods and Figure 1C,F in main text), the cutoff used for pruning the graphs corresponding to each data stream were different, directly determined, as shown in S2 Fig, by the choice of reporting rate and quantile.

```
## NULL
```

S3 Fig presents the clusters of cases identified using the different combinations of reporting rates and cutoffs. Lower reporting rates and higher quantiles led to higher cutoffs (see S2 Fig), and hence fewer edges removed at the pruning step. Therefore lower reporting rates and higher quantiles generally led to fewer, larger clusters. The most extreme scenarios considered here corresponded to i) a reporting rate of 50% and cutoffs corresponding to the 90% quantiles (bottomleft graph in S3 Fig) and ii) a reporting rate of 10% and cutoffs corresponding to the 98% quantiles (topright graph in S3 Fig). The former scenario led to a total of 89 clusters including 77 singletons, four pairs, and three clusters of respective sizes 3, 4 and 20. The latter led to a total of 8 clusters including 2 singletons, two pairs, and three clusters of respective sizes 7, 18 and 90. The clustering of cases in time, geographical space and genetic space, for the nine combinations of reporting rates and cutoffs are shown in S4 Fig, S5 Fig and S6 Fig respectively.

For each of the nine scenarios considered, we used the distribution of cluster sizes and the assumed reporting rate to estimate the reproduction number, as well as the total number of outbreaks (or clusters, i.e. separate introductions of rabies in the canine population) which occurred over the observation period, including the unobserved ones. The total number of outbreaks was translated into a rate of introductions by dividing the number of outbreaks by the duration of the monitoring period. Results are presented in S7 Fig. As discussed above, reporting rates and higher quantiles generally led to fewer, larger clusters, and hence higher estimates of the reproduction numbers, and lower estimates of the rate of introduction. Estimates of the reproduction number for the two extreme scenarios were 0.41 [95%CI 0.31, 0.52] and 0.98 [95%CI 0.93, 1.04]; the corresponding estimates of the yearly rate of introduction of rabies in the population were 6.4 [95%CI 4.5, 8.8] and 1.6 [95%CI 0.76, 3.2].

Real-time application

So far, we have illustrated our method by applying it retrospectively to analyse an outbreak of dog rabies in Central African Republic. However, our method would also be extremely useful if used during an ongoing outbreak, in particular to disentangle clusters of related cases from isolated cases due to separate introductions of the pathogen in the population. This could have a direct impact on control policies, providing information on whether to prioritise control measures aiming at reducing transmission or at reducing importations (or both).

In this section, we propose to mimic the real-time application of our method on the same rabies outbreak. We split the outbreak into three phases; each phase containing the same number of reported rabies cases with temporal, spatial and genetic data available (41 cases per time period). We apply our algorithm on the data restricted to the cases which appeared before the end of each of these phases. The 'early phase' analysis is restricted to cases before or on 24 April 2007; the 'early and peak phase' analysis further considers cases up to 06 June 2008. Finally, the full analysis (same as that presented in the main text) considers all cases. In this section, we assume a reporting rate of 20% and use a cutoff corresponding to the 95% quantile at the pruning step.

S8 Fig presents the epidemic curve available at the end of each of the three time periods considered, coloured according to the cluster allocation obtained at these three time points. There were very few changes in cluster allocation as more data got collected, with only 1 reclassification (out of 41 cases) between the early and the peak phase, and 3 reclassifications (out of 82 cases) between the peak phase and the end of the epidemic. This example therefore suggests that our method would provide useful insights into the epidemic dynamics in real-time.

Simulations

Simulation and reconstruction scenarios

We considered six simulation scenarios mimicking the transmission of rabies among dogs. Our baseline scenario was designed to closely mirror the transmission characteristics underlying the transmission of rabies in our dataset from Central African Republic. We then considered five variations of this scenario: a 'low', 'high' and 'perfect' reporting scenarios similar to the baseline scenario but with varying levels of reporting, and a 'low' and 'high' diversity scenarios, similar to our baseline but where the imported cases had pathogen genetic sequences respectively much more similar and much more different to one another (i.e. their most recent common ancestor was respectively more recent and more distant in the past). The scenarios are described in S1 Table.

For each simulation scenario, we used our method to reconstruct the clusters of cases linked by transmission, as well as re-estimate the reproduction number and the importation rate. In the reconstruction process, we used input distributions obtained by assuming the same mutation rate, genome length, serial interval and spatial kernel as in the simulation. For the baseline simulation scenario, we systematically varied the cutoffs and the assumed reporting rate, as described in S2 Table.

Simulation process

We adapted a simulation implemented in the function `simOutbreak` from the `outbreaker` R package, provided as supporting script. We also used simulation tools implemented in the R package `quicksim`, available from `https://github.com/thibautjombart/quicksim`.

We used a simple branching process for simulation. We considered a square of 15km $\times$ 15km. Each simulation was seeded with one initial case, randomly located in that square, and with symptom onset on day 0. The pathogen sequence for this index case was determined through random evolution from an ancestral sequence it diverged from for a fixed number of days determined by a simulation parameter (see S1 Table). We used a simple mutational model, in which every nucleotide of the ancestral genome ($n = 11,820$) mutates independently with a probability 1.62×10^{-6} per day, and mutations from and to every nucleotide are equiprobable. For each subsequent day t, newly infected cases emerging from local transmission as well as newly imported cases were simulated as follows.

The number of newly infected cases emerging from local transmission on day t was drawn from a Poisson distribution with mean $\lambda = R_0 \sum_{s=1}^{t} I_{t-s} w_s$, where R_0 is the basic reproduction number, I_{t-s} is the total number of cases that appeared at time t-s (both through local transmission and through importation) and w is the probability mass function of the serial interval. Each newly infected case was then allocated an infector, chosen among all cases who appeared in the past, with weights for cases who appeared at time $t - s$ equal to w_s. The location (x, y) of each newly infected case was then obtained by drawing x and y in independent normal distributions centered on the location of the infector. Whenever new coordinates would have been located outside the epidemics area, we implemented a 'mirror effect' which placed them back into the study area by symmetry with the existing borders.

The number of newly imported cases on day t was drawn from a Poisson distribution with mean the daily importation rate. The location of the newly imported cases was drawn at random within the square of 15kmx15km. The pathogen sequence for the newly imported cases were determined as for the index case, by modelling evolution from a common ancestral sequence.

The simulation was run for 8 years.

We assumed a constant reporting rate; observed cases were drawn from all cases according to a binomial distribution with probability given by the reporting rate.

Simulated datasets

We ran 200 simulations for each simulation scenario. For the baseline, low, high and perfect reporting scenarios, we used the same simulated epidemics but simulated different reporting levels.

The resulting simulated dataset sizes, i.e. the simulated number of observed cases for each simulation scenario, are shown in S9 Fig.

Reconstruction results

We quantify the ability of our method to correctly identify clusters of cases linked by transmission, through measuring 1) the true positive rate (TPR, proportion of pairs of cases actually linked by transmission who are inferred to be in the same outbreak cluster), i.e. the sensitivity of detecting a transmission link and 2) the true negative rate (TNR, proportion of pairs of cases not linked by transmission who are inferred to not be in the same outbreak cluster), i.e. the specificity of detecting a transmission link. We also compare the estimates of the reproduction number and importation rate to the values used in the simulation. A summary of the results of our simulation study are shown in the main text in Figure 5. S10 Fig shows the full estimates of the reproduction number and importation rate, i.e. central estimates as well as 95% confidence intervals. S11 Fig presents the root mean square error on the estimates of the reproduction number and the importation rate for all simulation and reconstruction scenarios considered, and suggests that estimates were precise across all scenarios.

Comparing different cutoffs

Our simulation study highlights the importance of adequately choosing the cutoff used at the pruning step to yield a high sensitivity and specificity of detecting outbreak clusters. We applied our method to the same set of baseline simulations but with different cutoffs, corresponding respectively to the 50%, 90%, 95%, $95\%^{1/3} = 98\%$ and 99.9% quantiles of the input distributions. For each simulation, we computed the TPR (or sensitivity) and the TNR (or specificity) obtained with each cutoff, and we recorded which of the cutoffs yielded the best performance, i.e. the highest average between sensitivity and specificity. Results, presented in S12 Fig, suggest that for rabies and with a reporting rate of 20%, a cutoff corresponding to the 95% quantiles is optimal.

Although impractical to plot for all 200 simulations together, for a single simulation, we can plot a ROC (Receiver Operating Characteristic) curve to graphically illustrate the trade-off between sensitivity and specificity and the cutoff maximising the combination of the two.

We selected, among the 200 baseline simulations, the only simulated dataset which happened to have the same size as our rabies dataset, i.e. 151 observed cases. For this dataset, we applied our method with 9 cutoffs, defined by the 50%, 75%, 90%, 95%, $95\%^{1/3} = 98.3\%$, 99%, 99.9%, 99.95%, and 99.99% quantiles. The corresponding ROC curve is shown in S13 Fig. In this specific simulation, the 95% cutoff is the optimal one; but as shown in S12 Fig this can vary from one simulation to the other.

Computing time

For all simulation scenarios, we recorded the computing time associated with the identification of outbreak clusters under the 'control' reconstruction scenario (see 'Simulation and reconstruction scenarios' section, S1 Table and S2 Table for a definition of the simulation and reconstruction scenarios). Pooling results from all six simulation scenarios together, we then considered linear regressions between the computing time and various powers of the simulated dataset size. We considered powers from 0.5 to 4, with 0.1 increments, and measured the associated adjusted R^2, which was maximal for a power of 2.2, with an adjusted R^2 of 0.985. These results are presented in S14 Fig.

References

1. Campbell F, Strang C, Ferguson N, Cori A, Jombart T. When are pathogen genome sequences informative of transmission events? PLoS Pathogens. 2018;14(2):e1006885.

2. Farrington CP, Kanaan MN, Gay NJ. Branching process models for surveillance of infectious diseases controlled by mass vaccination. Biostatistics. 2003;4(2):279–295. doi:10.1093/biostatistics/4.2.279.

3. Waxman D, Nouvellet P. Sub- or supercritical transmissibilities: Symmetry in outbreak properties of a disease when conditioned on extinction. Journal of Theoretical Biology. under review;.

4. Hampson K, Dushoff J, Cleaveland S, Haydon DT, Kaare M, Packer C, et al. Transmission dynamics and prospects for the elimination of canine rabies. PLoS Biology. 2009;7(3):e1000053.

5. Svensson O. A note on generation times in epidemic models. Mathematical Biosciences. 2007;208(1):300 – 311. doi:http://dx.doi.org/10.1016/j.mbs.2006.10.010.

6. Bourhy H, Nakouné E, Hall M, Nouvellet P, Lepelletier A, Talbi C, et al. Revealing the micro-scale signature of endemic zoonotic disease transmission in an African urban setting. PLoS Pathogens. 2016;12(4):e1005525.
