# Supplementary figures and images for: A graph-based evidence synthesis approach to detecting outbreak clusters: An application to dog rabies
Source: PLoS Comput Biol. 2018 Dec 17;14(12):e1006554. doi: 10.1371/journal.pcbi.1006554 (PMC6312344; doi:10.1371/journal.pcbi.1006554)

**Delays distribution**

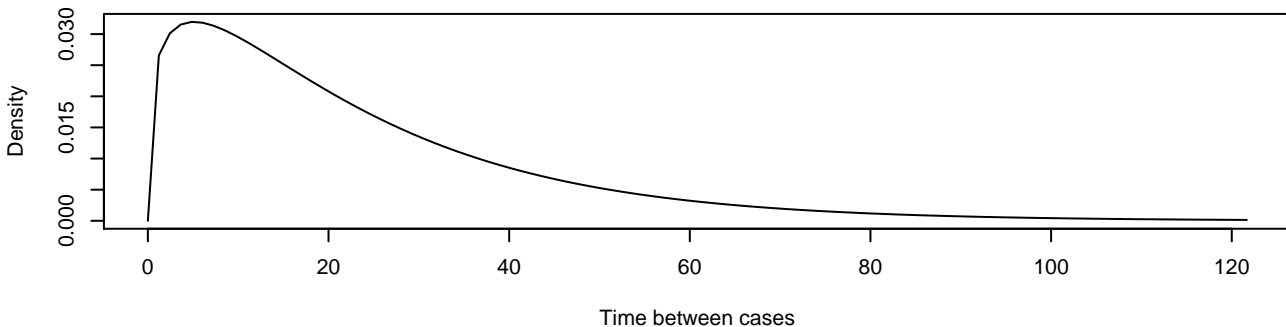

**Dispersal distance distribution**

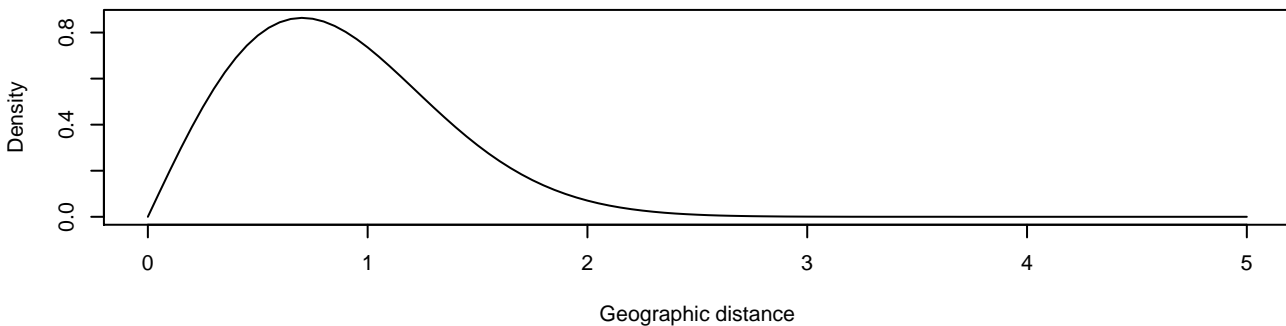

**Genetic signature distribution**

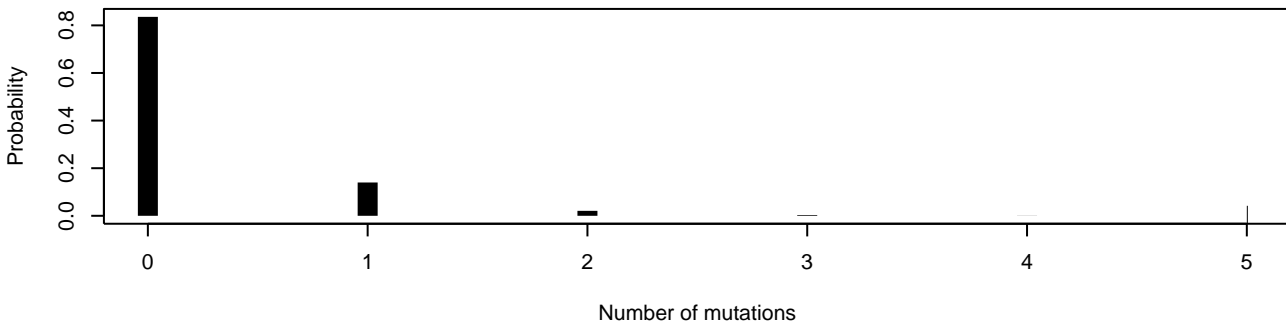

Supplement: S1 Fig — Top: Distribution of the serial interval in days (here defined as the time between reporting of a case and its infector). Middle: Distribution of the spatial kernel in kilometers (the distance between a case and its infector). Bottom: Distribution of the number of mutations between the whole genome sequence of the pathogen sampled from a case and its infector. (PDF) [file pcbi.1006554.s002.pdf]

**Cutoff: 90 %**

**Cutoff: 95 %**

**Cutoff: 98 %**

**Reporting: 10 %**

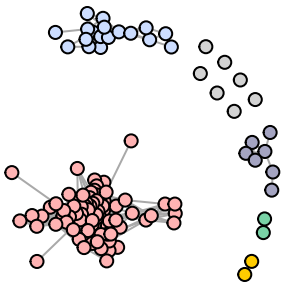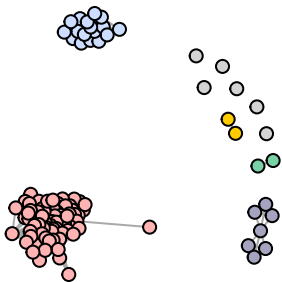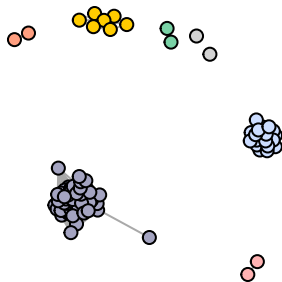

**Reporting: 20 %**

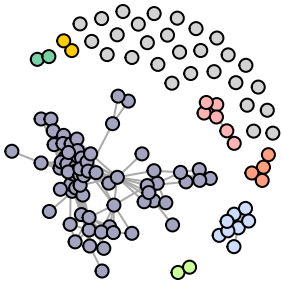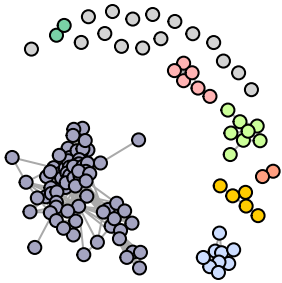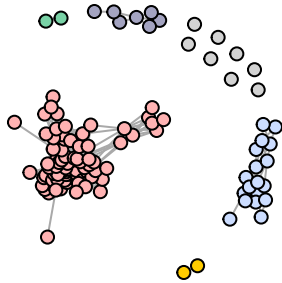

**Reporting: 50 %**

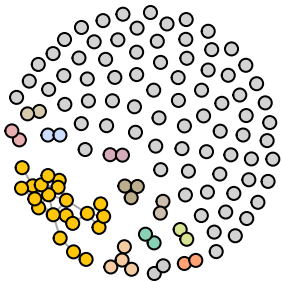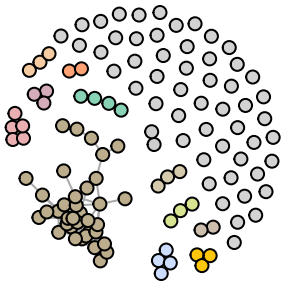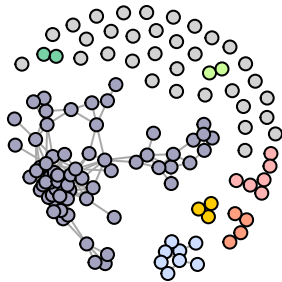

Supplement: S3 Fig — Graph representation of clusters of cases in the rabies outbreak, obtained from temporal, spatial and genetic data together, and using different assumptions on the reporting rate (10%, top row; 20%, middle row; and 50%, bottom row) and cutoff for pruning (corresponding to the 90% quantile, left column; 95% quantile, middle column; and 98% quantile; right column). The colours of the nodes (i.e. cases) correspond to the cluster they belong to according to the analysis using a given combination of reporting rate and cutoff. Grey indicates singletons. (PDF) [file pcbi.1006554.s004.pdf]

**Cutoff: 90 %**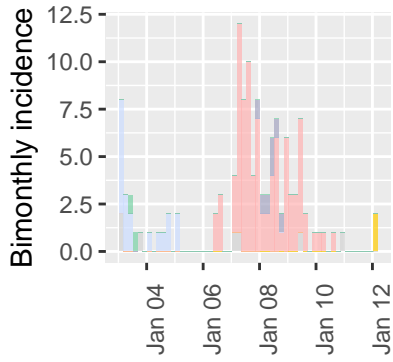**Cutoff: 95 %**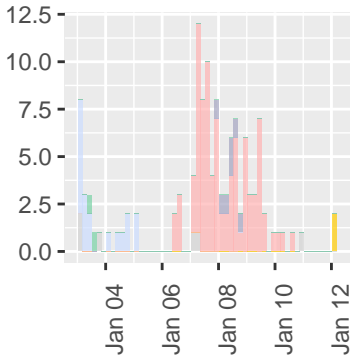**Cutoff: 98 %**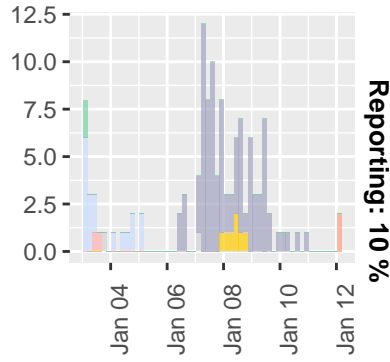**Reporting: 10 %**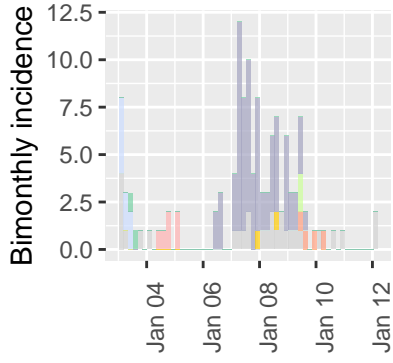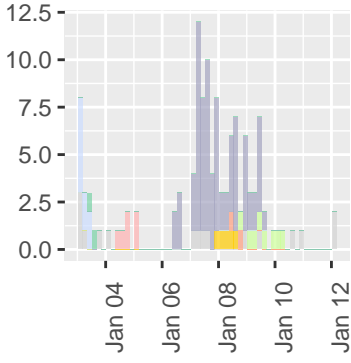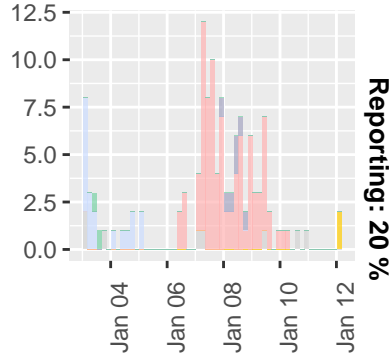**Reporting: 20 %**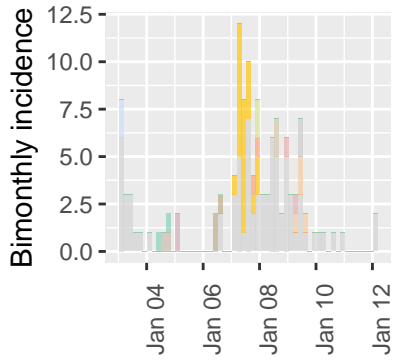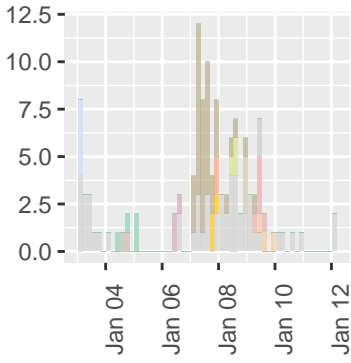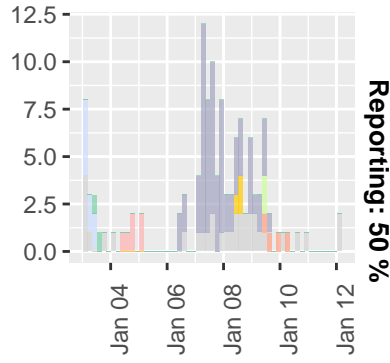**Reporting: 50 %**

Supplement: S4 Fig — Visualization of the clusters of cases of rabies in time, using different assumptions on the reporting rate (10%, top row; 20%, middle row; and 50%, bottom row) and cutoff for pruning (corresponding to the 90% quantile, left column; 95% quantile, middle column; and 98% quantile; right column). The colours correspond to the cluster cases are inferred as belonging to according to the analysis using a given combination of reporting rate and cutoff. Grey indicates singletons. (PDF) [file pcbi.1006554.s005.pdf]

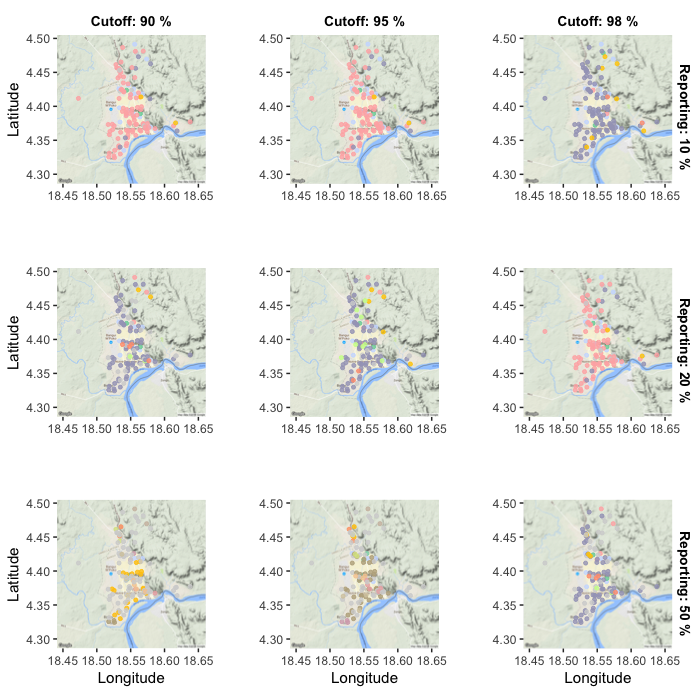

Supplement: S5 Fig — Visualization of the clusters of cases of rabies in space, using different assumptions on the reporting rate (10%, top row; 20%, middle row; and 50%, bottom row) and cutoff for pruning (corresponding to the 90% quantile, left column; 95% quantile, middle column; and 98% quantile; right column). The colours correspond to the cluster cases are inferred as belonging to according to the analysis using a given combination of reporting rate and cutoff. Grey indicates singletons. (PNG) [file pcbi.1006554.s006.png]

Estimated rate  
of importation (per year)

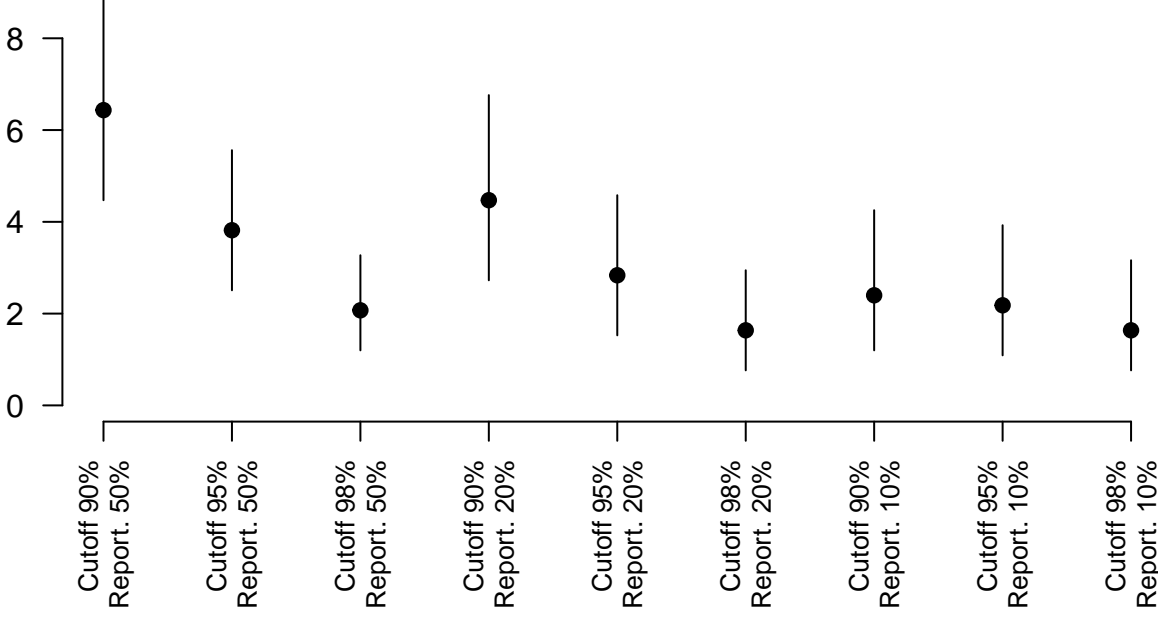

Estimated R

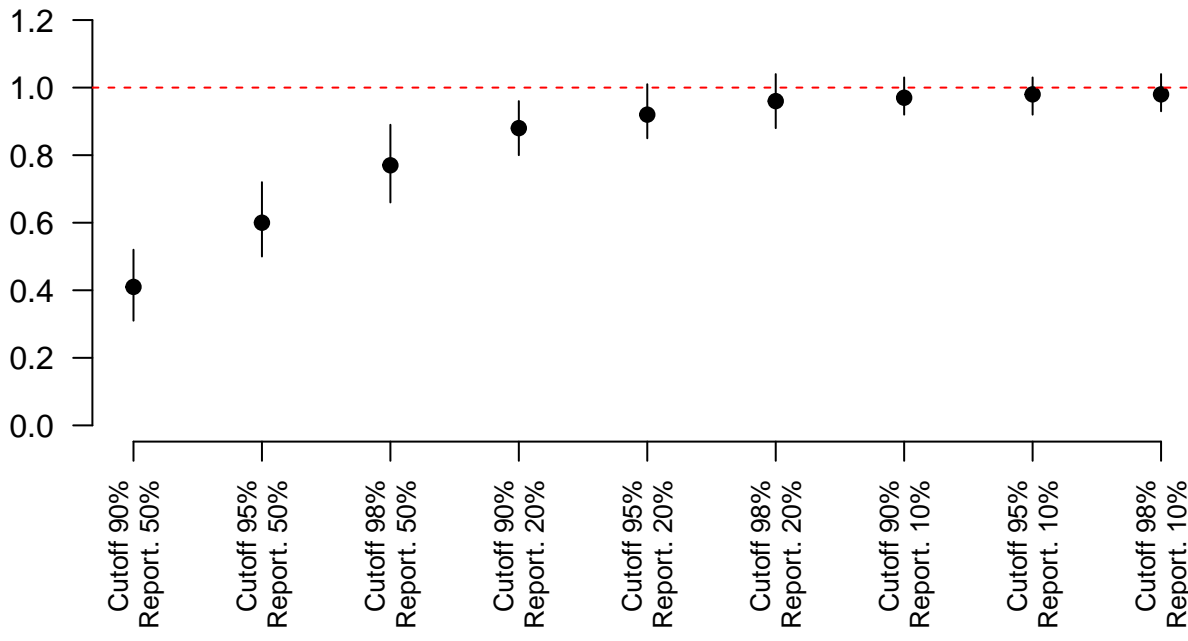

Supplement: S7 Fig — Estimated reproduction number (top) and yearly rate of importation of rabies in the population (bottom), for different assumptions on the reporting rate (10%, 20% or 50%) and cutoff for pruning (corresponding to the 90%, 95% or 98% quantile). The dots correspond to the maximum likelihood estimates and the vertical bars show the 95% confidence intervals around these. (PDF) [file pcbi.1006554.s008.pdf]

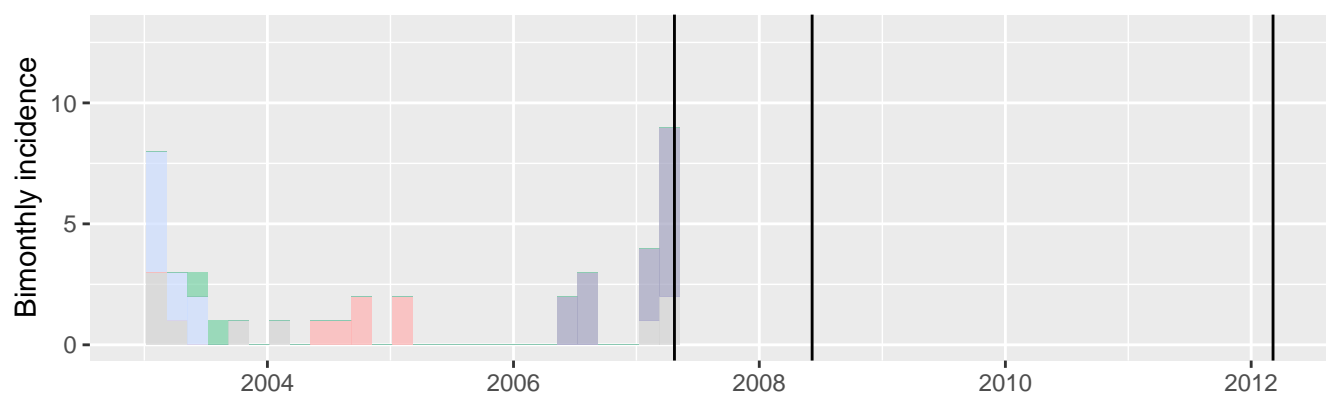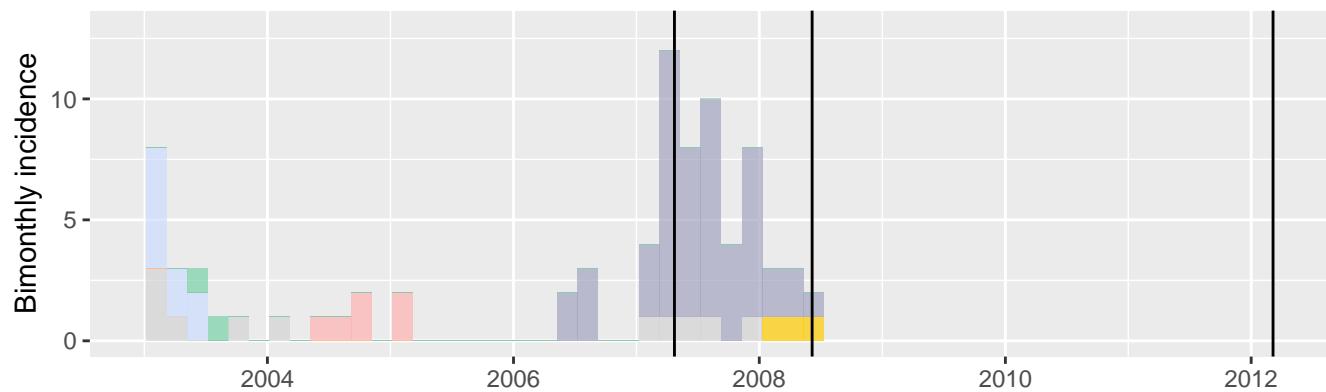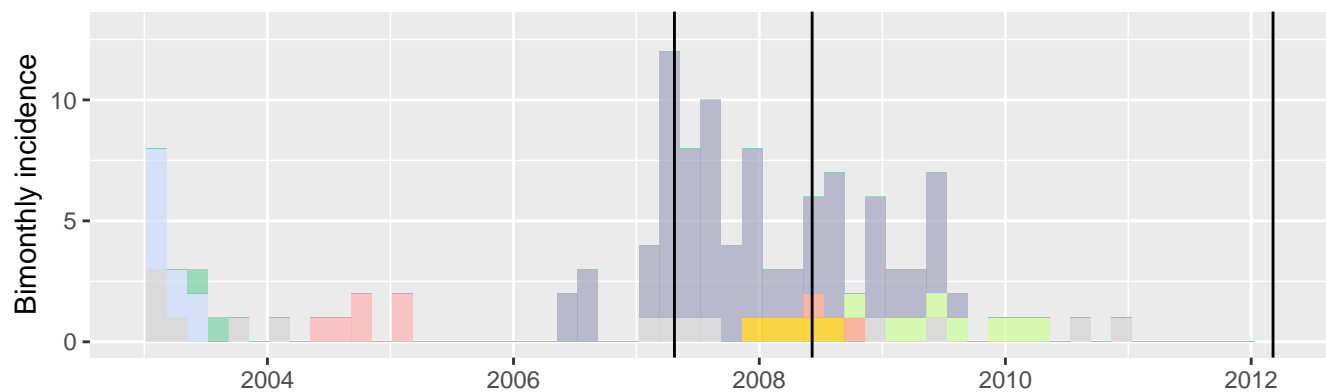

Supplement: S8 Fig — Visualization of the clusters of cases of rabies in time, inferred at different times in the outbreak (top: 24 April 2007; middle: 06 June 2008; bottom: 06 March 2012), assuming a reporting rate of 20% and using a cutoff for pruning corresponding to the 95% quantile. The colours correspond to the cluster cases are inferred as belonging to according to the analysis performed at each time point. Grey indicates singletons. The vertical lines show the three dates at which the algorithm was run. (PDF) [file pcbi.1006554.s009.pdf]

Number of cases in simulated dataset

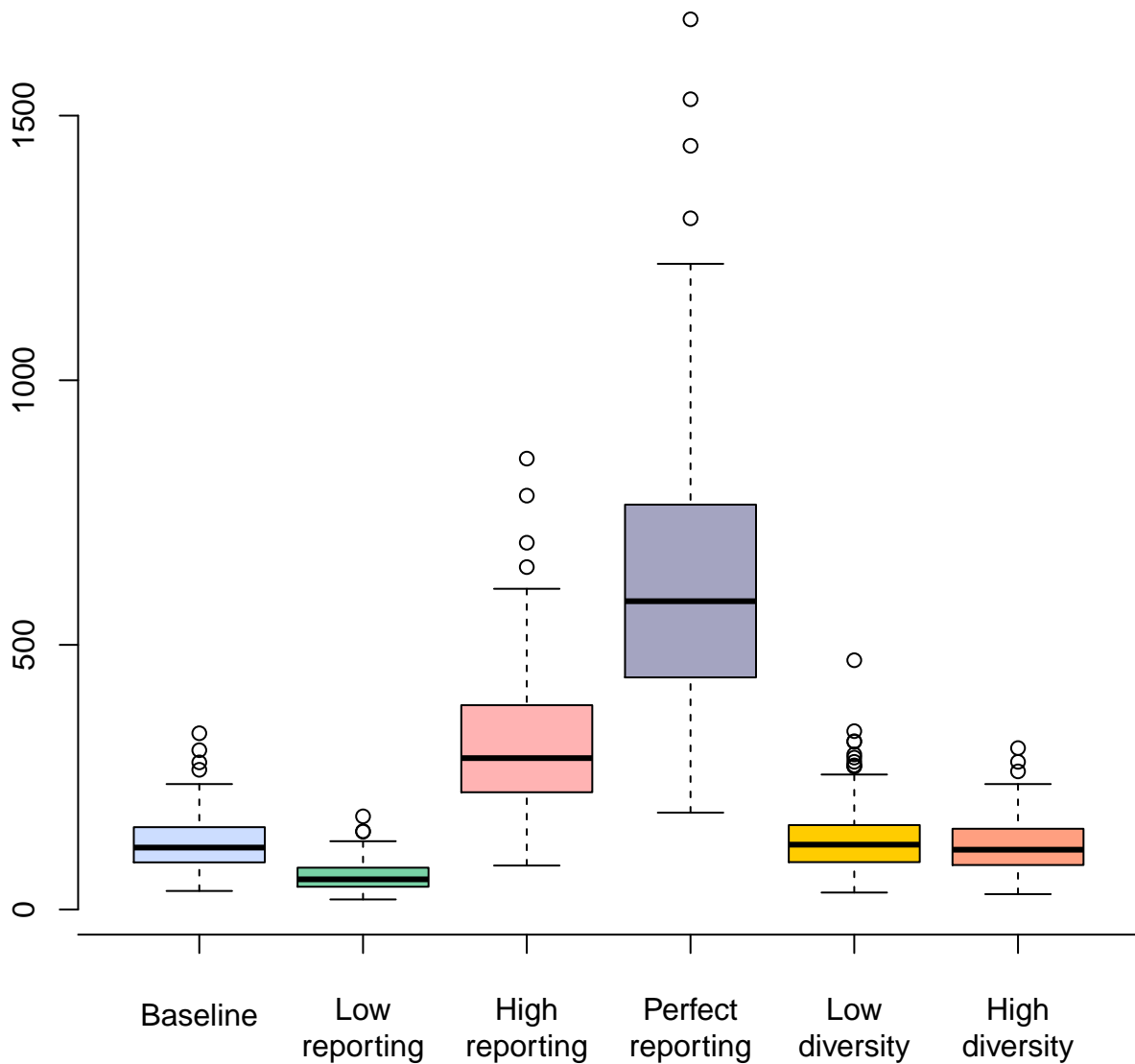

Supplement: S9 Fig — (PDF) [file pcbi.1006554.s010.pdf]

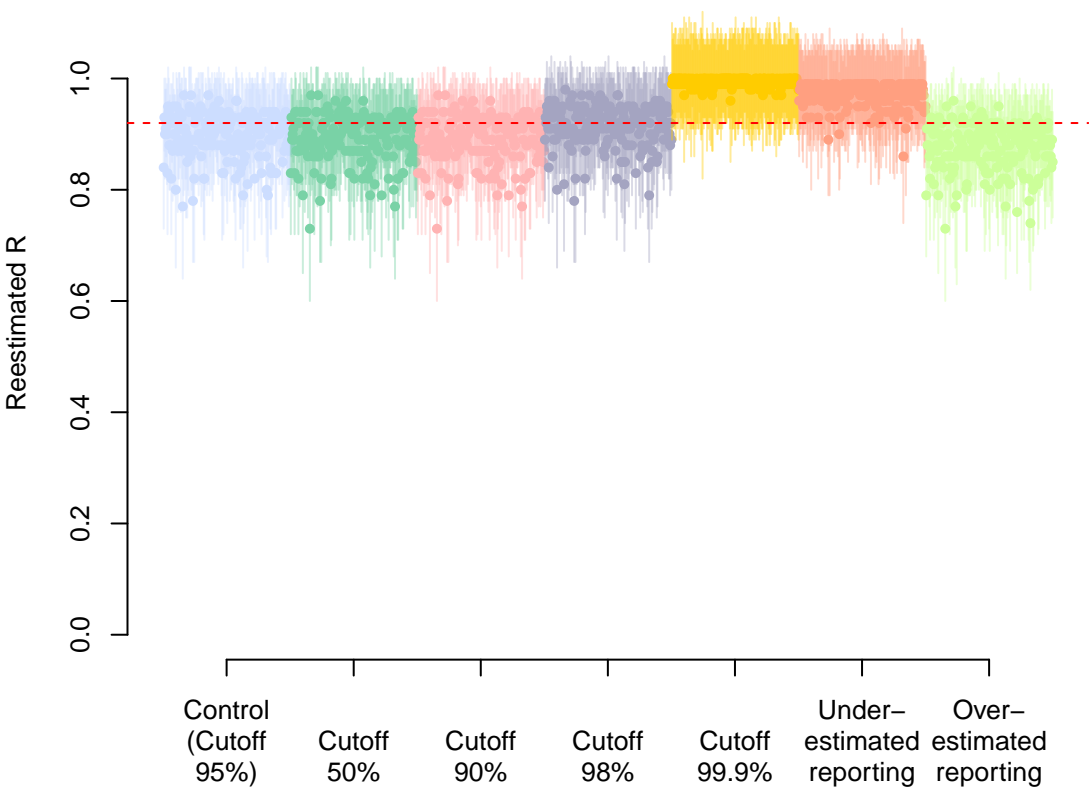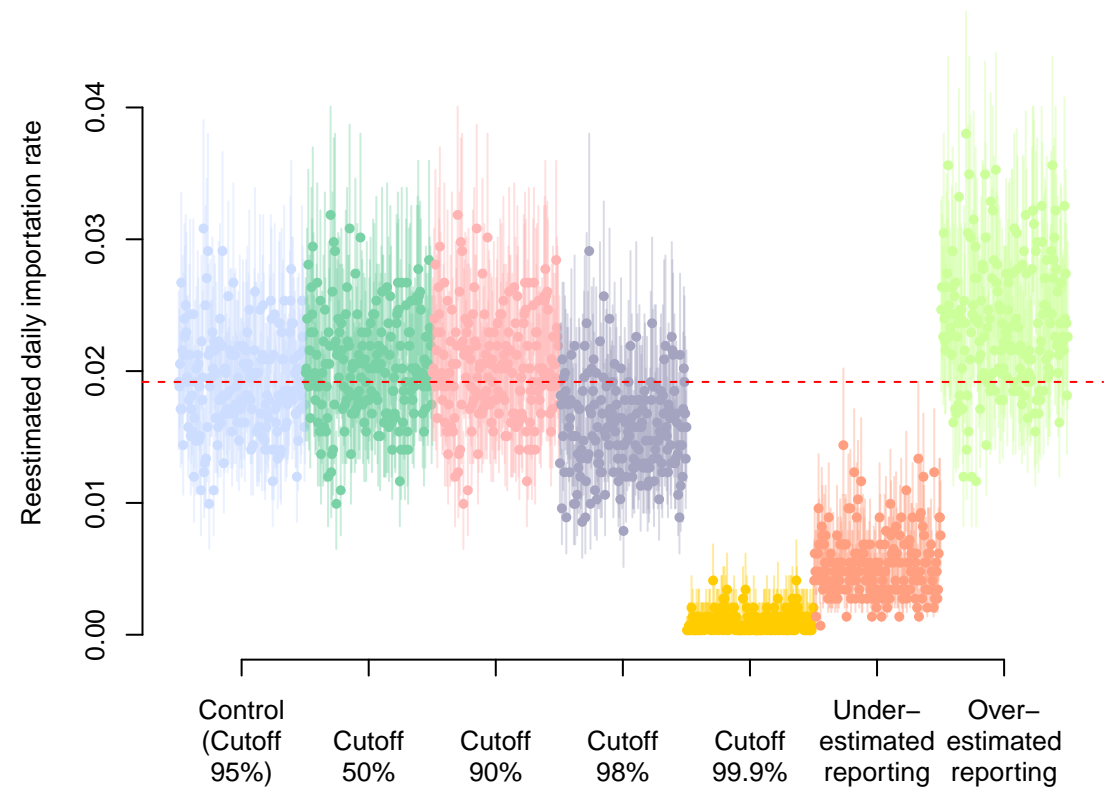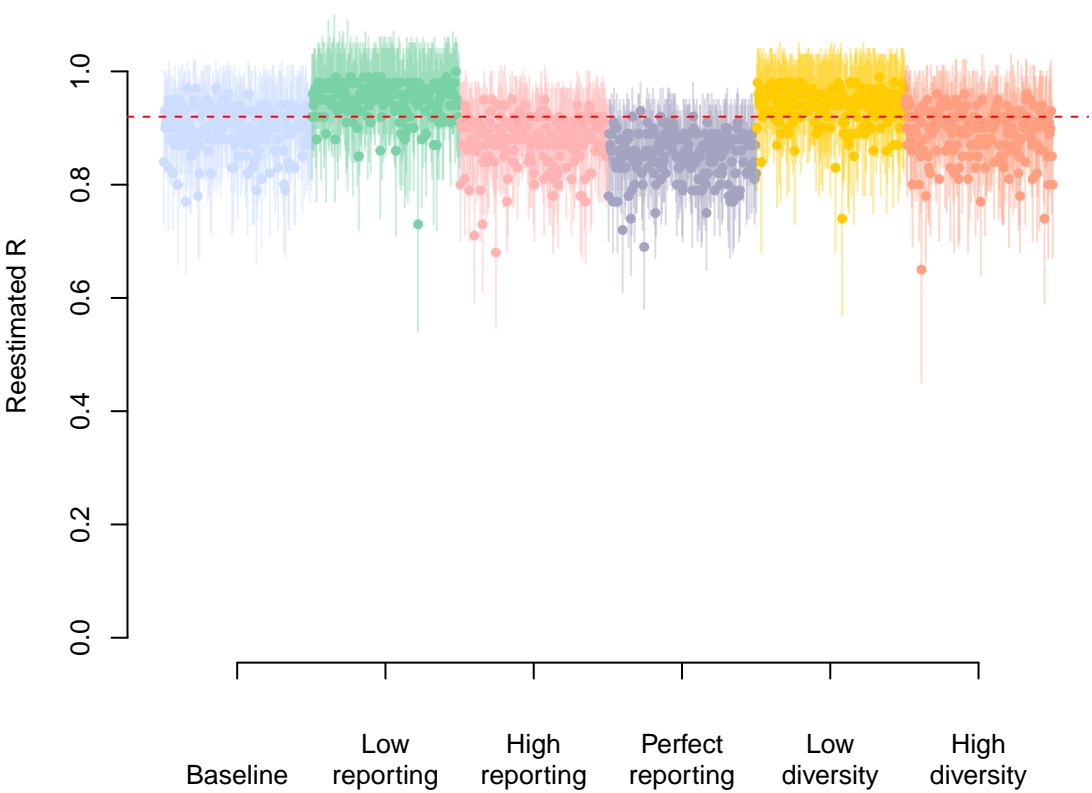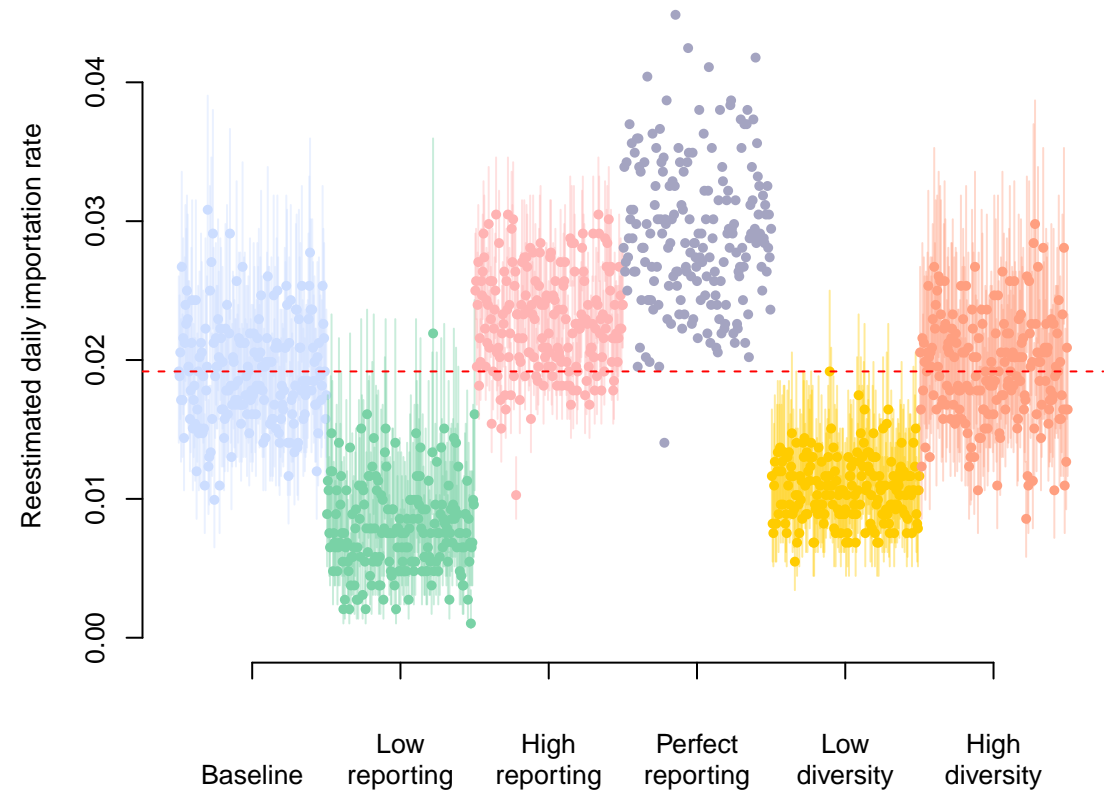

Supplement: S10 Fig — Reestimated reproduction number (left) and importation rate (right), for each of the seven reconstruction scenarios applied to the 'baseline' simulation scenario (top) and for the 'control' reconstruction scenario applied to each of the six simulation scenarios (bottom). See S1 Text, S1 Table and S2 Table for a definition of the simulation and reconstruction scenarios. The dots show the central estimates for each simulation; the vertical bars show the 95% confidence intervals. The red horizontal dashed lines show the values used in the simulation. Note in the perfect reporting scenario, there is no uncertainty on the estimate of the importation rate. (PDF) [file pcbi.1006554.s011.pdf]

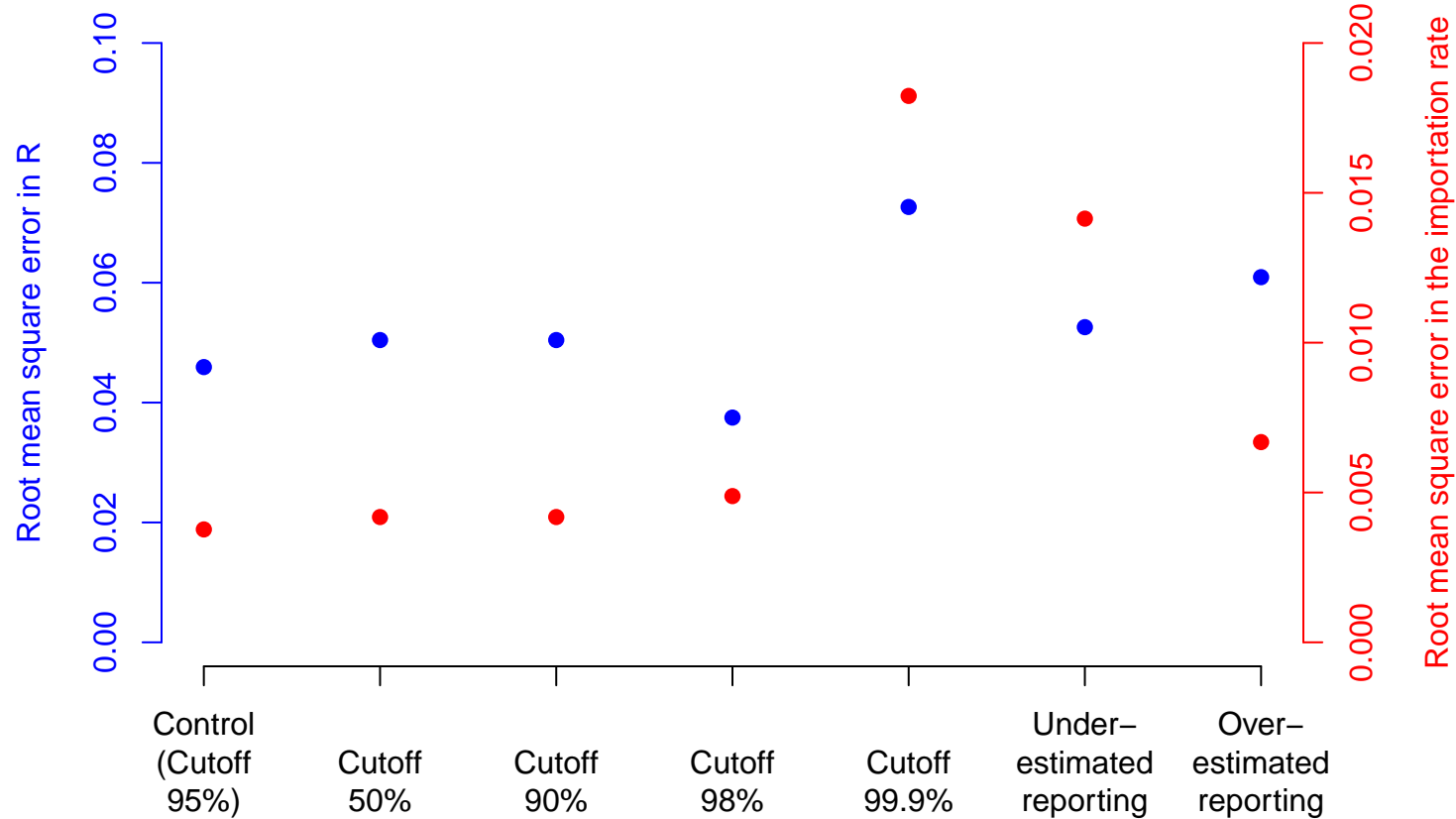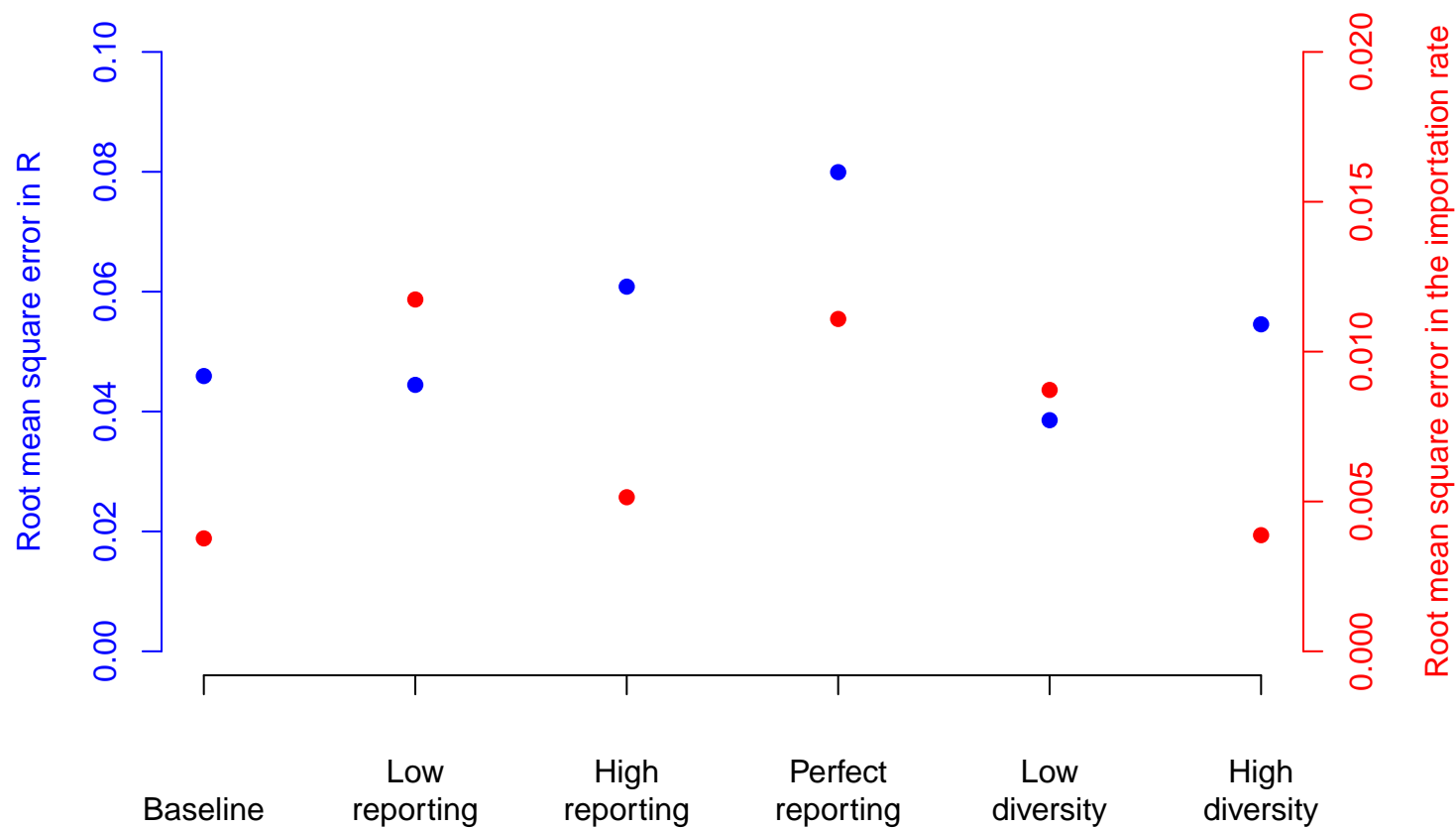

Supplement: S11 Fig — Root mean square error in the estimated reproduction number (R, left axis) and in the estimated importation rate (right axis), for each of the seven reconstruction scenarios considered (see S1 Text and S2 Table for a definition of the reconstruction scenarios). (PDF) [file pcbi.1006554.s012.pdf]

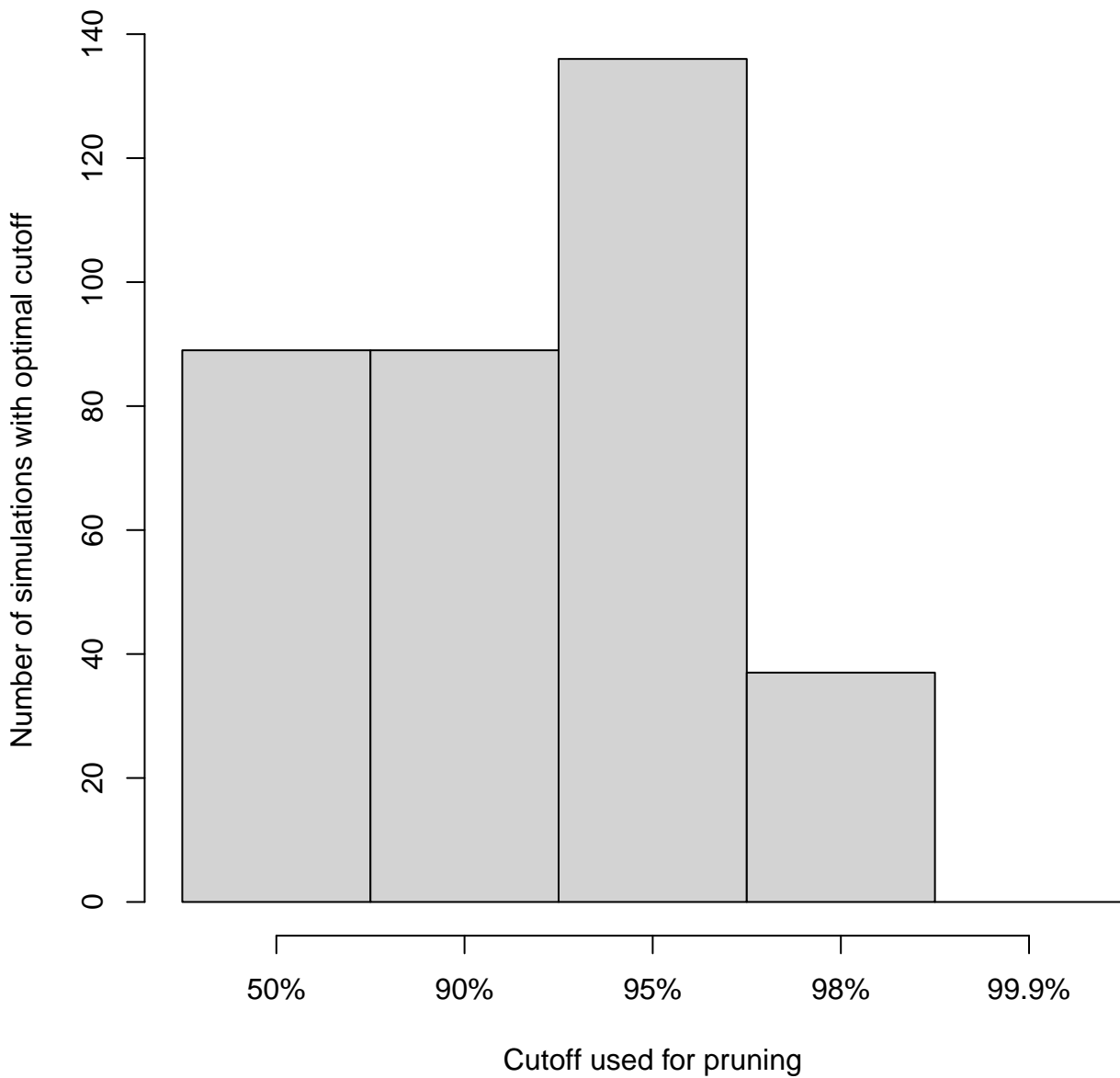

Supplement: S12 Fig — Note that for certain simulations, several cutoffs yielded the same performance, so a single simulation may be contributing to several cutoffs. (PDF) [file pcbi.1006554.s013.pdf]

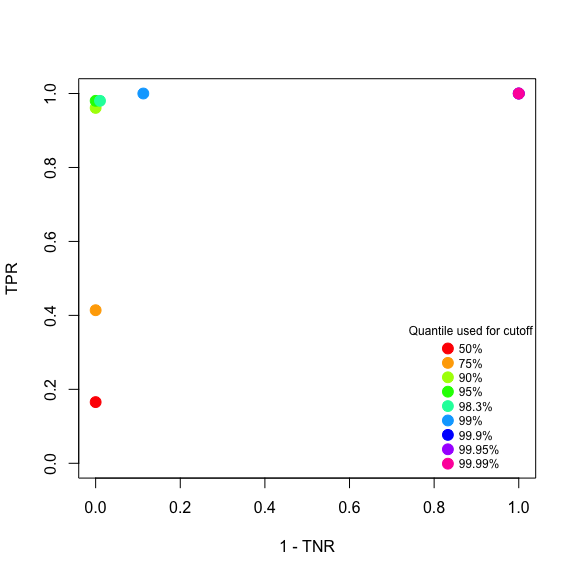

Supplement: S13 Fig — The simulated dataset was selected, among the 200 baseline simulations, as the only simulated dataset which happened to have the same size as our rabies dataset, i.e. 151 observed cases. TPR: True Positive Rate; TNR: True Negative Rate. (PNG) [file pcbi.1006554.s014.png]

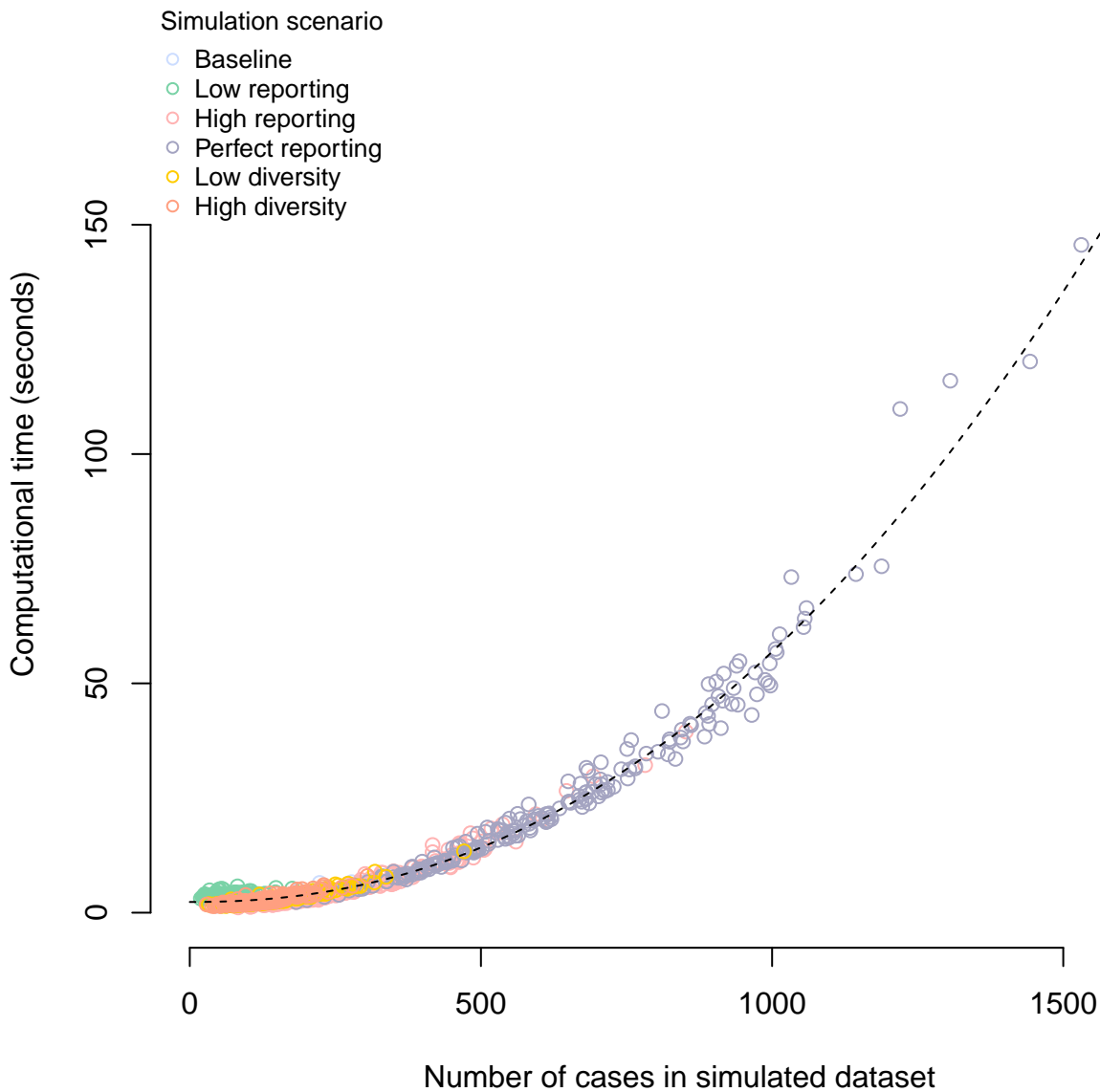

Supplement: S14 Fig — The computing time encompasses the time required to 1) compute the pairwise distances in time, geographical space, and genetic space, 2) calculate the cutoffs associated with the quantile chosen (here 95%) and the assumed reporting rate (here 20%), and 3) construct, prune, and merge the graphs to define clusters of cases linked by local transmission. Note the computing time only measures the time required to perform the reconstruction, not the simulation. The dashed line represents y = 2.33 + 1.37 * 10−5 x2.2. (PDF) [file pcbi.1006554.s015.pdf]
