# Supplementary material for: A graph-based evidence synthesis approach to detecting outbreak clusters: An application to dog rabies
Source: PLoS Comput Biol. 2018 Dec 17;14(12):e1006554. doi: 10.1371/journal.pcbi.1006554 (PMC6312344; doi:10.1371/journal.pcbi.1006554)
Supplement: S2 Fig — Distribution of pairwise temporal (left), spatial (middle) and genetic (right) distances for rabies. The temporal distance is defined as the time between sampling of the pathogen in cases. The spatial distance is defined as the Euclidean distance between the locations of cases. The genetic distance is defined as the number of Single Nucleotide Polymorphisms (SNPs) between the Whole Genome Sequences (WGS) sampled from cases. The grey histograms show the observed pairwise distances between any two cases reported in the Bangui outbreak. The solid black lines show the assumed distribution of distances between a case and its closest observed ancestry, given an assumed reporting rate of 10% (top row), 20% (middle row) and 50% (bottom row); note that the distributions have been rescaled to fit on the same graph as the histograms. The vertical lines show the cutoffs corresponding to the 90% (yellow), 95% (orange) and 98% (red) quantiles of these distributions. For a given data stream and a given choice of reporting rate and cutoff, pairs of cases with observed distance above the cutoff are considered not connected, and the corresponding graph edges are removed at the pruning step (see Fig 1 in main text). (PDF) [file pcbi.1006554.s003.pdf]

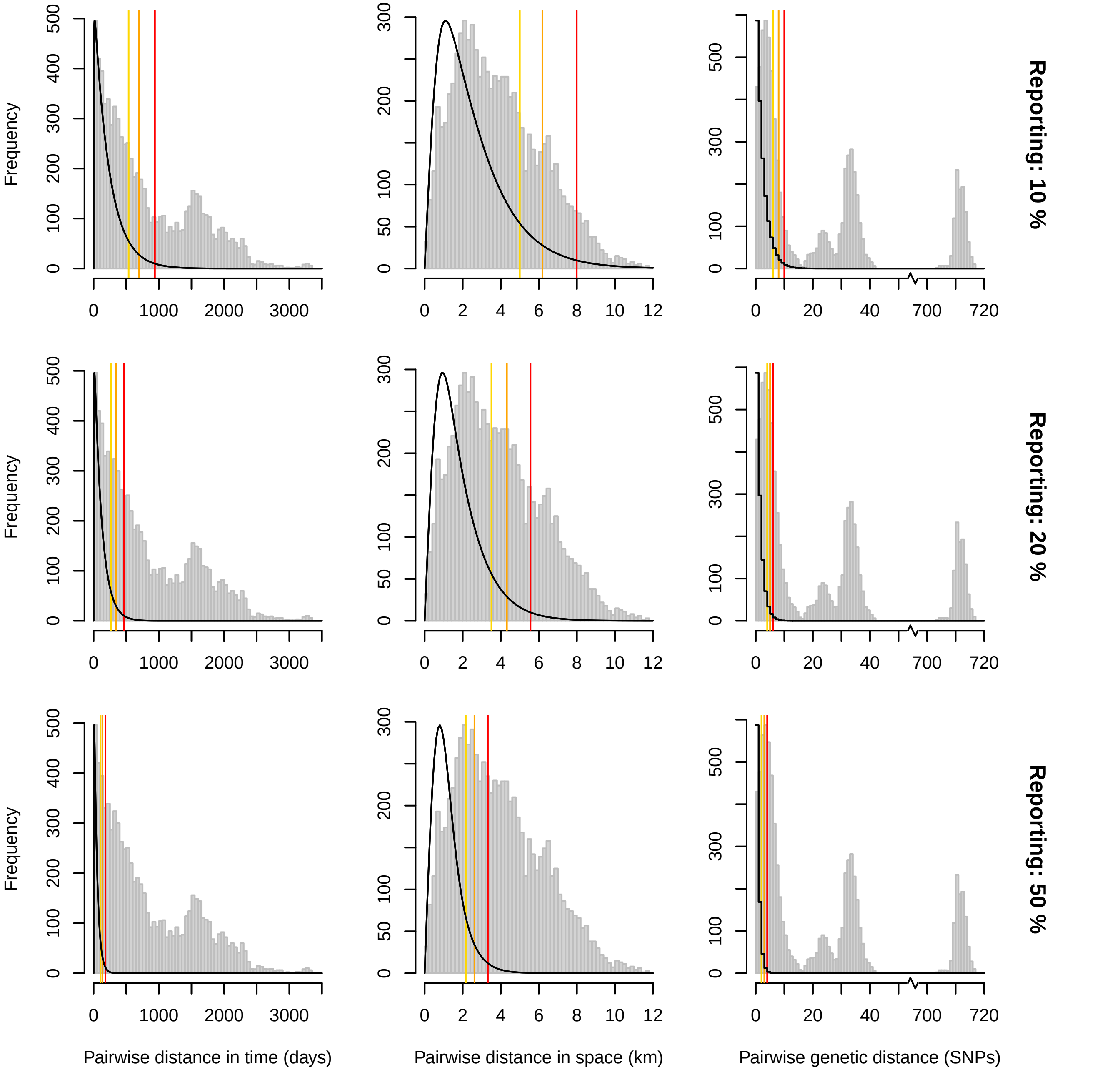
Frequency
Frequency
Frequency
Reporting: 10 %
Reporting: 20 %
Reporting: 50 %
0
100
200
300
400
500
0
50
100
200
300
0
1000
2000
3000
0
2
4
6
8
10
12
0
20
40
700
720
Pairwise distance in time (days)
Pairwise distance in space (km)
Pairwise genetic distance (SNPs)
