# Supplementary material for: A graph-based evidence synthesis approach to detecting outbreak clusters: An application to dog rabies
Source: PLoS Comput Biol. 2018 Dec 17;14(12):e1006554. doi: 10.1371/journal.pcbi.1006554 (PMC6312344; doi:10.1371/journal.pcbi.1006554)
Supplement: S1 Table — MRCA: most recent common ancestor; -: as baseline; sd: standard deviation; *: https://www.ncbi.nlm.nih.gov/nuccore/JQ685977.1 (PDF) [file pcbi.1006554.s016.pdf]

Simulation scenario	Mutation rate (per site per day)	Genome length (base pairs)	Time to MRCA of all imported cases	Reproduction number	Serial interval: mean (sd) (days)	Sd of spatial kernel	Importation rate (introductions per year)	Reporting rate
**Baseline**	1.62×10^{-6} (Bourhy et al.)	$11,820^{*}$	1 year	0.92 (our estimate for rabies)	23.6 (20.9) (Hampson et al.)	0.70km (Hampson et al.)	7 (Bourhy et al.)	20% (Bourhy et al.)
**Low reporting**	-	-	-	-	-	-	-	10% (lower bound in Bourhy et al.)
**High reporting**	-	-	-	-	-	-	-	50% (upper bound in Bourhy et al.)
**Perfect reporting**	-	-	-	-	-	-	-	100%
**Low diversity**	-	-	6 months	-	-	-	-	-
**High diversity**	-	-	5 years	-	-	-	-	-

Table S1: Simulation scenarios. MRCA: most recent common ancestor; -: as baseline; sd: standard deviation; *: `https://www.ncbi.nlm.nih.gov/nuccore/JQ685977.1`
