# Supplementary material for: A graph-based evidence synthesis approach to detecting outbreak clusters: An application to dog rabies
Source: PLoS Comput Biol. 2018 Dec 17;14(12):e1006554. doi: 10.1371/journal.pcbi.1006554 (PMC6312344; doi:10.1371/journal.pcbi.1006554)
Supplement: S2 Table — (PDF) [file pcbi.1006554.s017.pdf]

**Reconstruction scenario**	**Quantile used for cutoff definition**	**Reporting rate**
**Control**	0.95	As in simulation
**50% cutoffs**	0.50	As in simulation
**90% cutoffs**	0.90	As in simulation
**98% cutoffs**	$0.95^{1/3}$	As in simulation
**99.9% cutoffs**	0.999	As in simulation
**Underestimated reporting**	0.95	0.1
**Overestimated reporting**	0.95	0.4

Table S2: Reconstruction scenarios
