# Supplementary material for: A graph-based evidence synthesis approach to detecting outbreak clusters: An application to dog rabies
Source: PLoS Comput Biol. 2018 Dec 17;14(12):e1006554. doi: 10.1371/journal.pcbi.1006554 (PMC6312344; doi:10.1371/journal.pcbi.1006554)
Supplement: S6 Fig — The two clades referred to as A (top) and B (bottom) in the main text are represented separately for more readability (clades are distant by more than 700 nucleotides, masking all other diversity). The full phylogeny was obtained by Neighbour-Joining on Hamming distances. Results are shown for various combinations of the reporting rate (10%, top row; 20%, middle row; and 50%, bottom row) and cutoff for pruning (corresponding to the 90% quantile, left column; 95% quantile, middle column; and 98% quantile; right column). The colours correspond to the cluster cases are inferred as belonging to according to the analysis using a given combination of reporting rate and cutoff. Grey indicates singletons. (PDF) [file pcbi.1006554.s007.pdf]

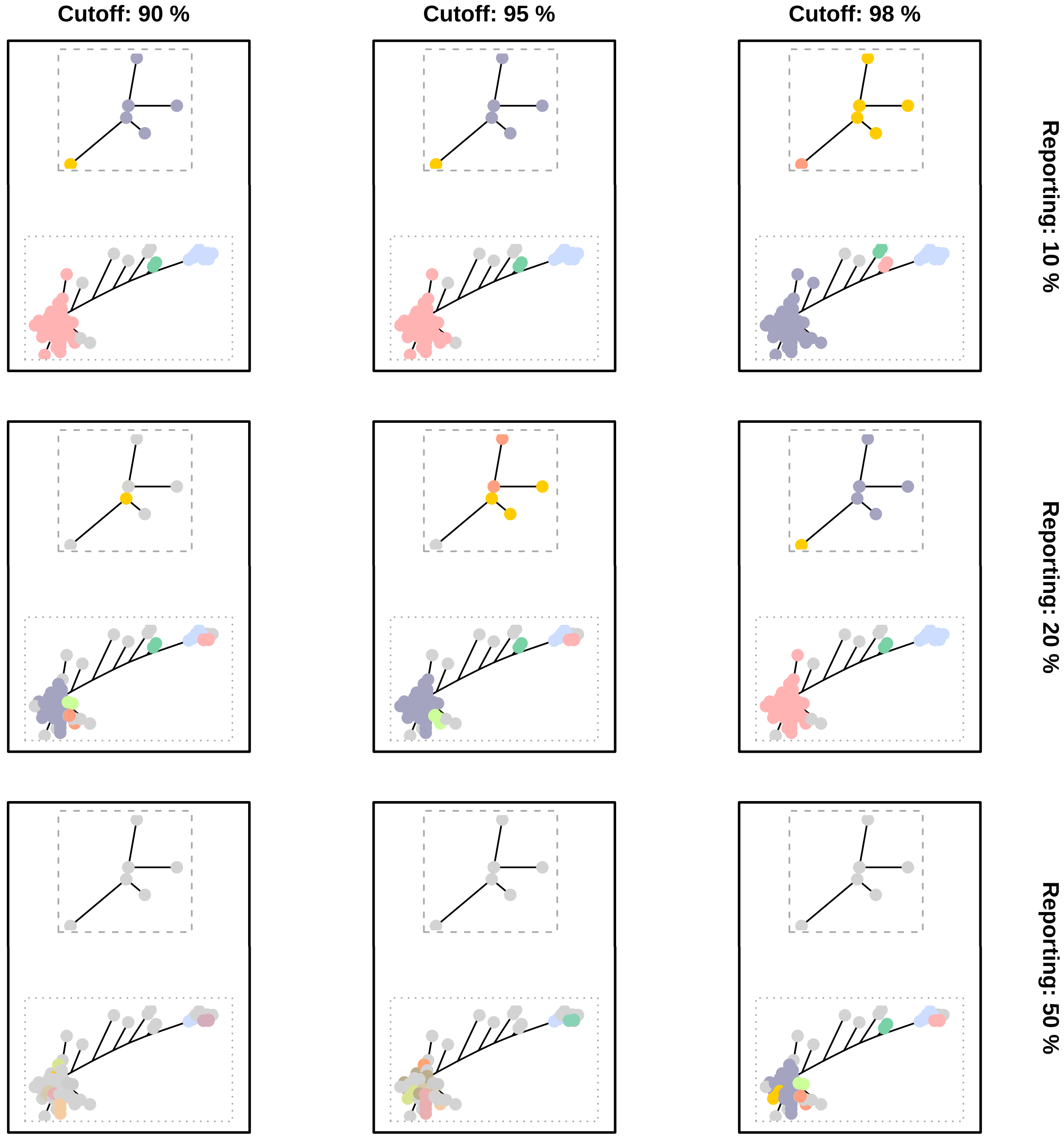

Cutoff: 90 %
Cutoff: 95 %
Cutoff: 98 %
Reporting: 10 %
Reporting: 20 %
Reporting: 50 %
